# Ocean warming events resilience capability in underwater computing platforms

**DOI:** 10.1038/s41598-024-54050-8

**Published:** 2024-02-15

**Authors:** A. A. Periola, A. A. Alonge, K. A. Ogudo

**Affiliations:** 1 Electrical, Electronic, and Computer Engineering, Cape Peninsula University of Technology, Cape Town, South Africa; 2 Electrical and Electronic Engineering Technology, University of Johannesburg, Johannesburg, South Africa

**Keywords:** Underwater data centers, Operational duration, Power usage effectiveness, Marine heat waves, Underwater seismic events, Data, Mobile computing systems, Mobile networks, Cloud computing, Engineering, Mathematics and computing

## Abstract

Underwater data centers (UDCs) use the ocean’s cold-water resources for free cooling and have low cooling costs. However, UDC cooling is affected by marine heat waves, and underwater seismic events thereby affecting UDC functioning continuity. Though feasible, the use of reservoirs for UDC cooling is non–scalable due to the high computing overhead, and inability to support continuity for long duration marine heat waves. The presented research proposes a mobile UDC (capable of migration) to address this challenge. The proposed UDC migrates from high underwater ground displacement ocean regions to regions having no or small underwater ground displacement. It supports multiple client underwater applications without requiring clients to develop, deploy, and launch own UDCs. The manner of resource utilization is influenced by the client’s service level agreement. Hence, the proposed UDC provides resilient services to the clients and the requiring applications. Analysis shows that using the mobile UDC instead of the existing reservoir UDC approach enhances the operational duration and power usage effectiveness by 8.9–48.5% and 55.6–70.7% on average, respectively. In addition, the overhead is reduced by an average of 95.8–99.4%.

## Introduction

Data centers play an important role in content access via computing and communication networks. The operation of data centers faces challenges such as high operational costs arising from the necessity of server cooling. The need to reduce the cost of cooling is an important challenge with regard to the operation of data centers^1–6^. The use of a significant amount of energy in cooling also results in a poor data center power usage effectiveness (PUE). Existing research has proposed approaches aimed at jointly reducing cooling costs and enhancing the PUE^7–9^. In addition, research has identified that siting data centers in non–terrestrial locations serve to reduce cooling costs. Such non–terrestrial locations have free cooling resources. Examples of such locations are stratosphere^10^, outer space^11–13^, and the underwater environment^14–16^. The underwater environment is capable of reducing data center cooling costs. The freely available cooling water in the underwater environment results in a reduced cooling cost. However, using underwater data centers (UDCs) faces challenges due to the variability in cooling water availability. The variability arises due to marine heat waves and is addressed in^16^. However, additional challenges arise in the underwater environment that affect UDC cooling performance. The concerned challenges arise due to the harsh context presented by the underwater environment.

An important challenge that needs to be considered in this regard is that of underwater volcanic activity^17,18^. The occurrence of underwater seismic events i.e., underwater volcanic activity is a challenge that significantly affects UDC performance. This is because they cause significant underwater ground displacement. Therefore, it is necessary to consider the underwater ground water displacement as an important parameter in UDC planning, deployment, and utilization. However, the consideration of UDCs in research^14–16^ and in enterprise deployment^19–21^ is yet to consider the occurrence of underwater volcanic activity.

However, it is important to consider the influence of underwater volcanic activity on UDC continued cooling and functionality. The consideration is important to ensure continued UDC functionality in applications such as communication networks and systems that derive low latency content access when UDCs are deployed. The research being presented aims to design a UDC that is robust to the occurrence of underwater volcanic activity. The research contributions in this paper are given as:

1. First, the paper proposes and presents an underwater sensor network (USN) hosting sensor that detect underwater ground displacement. The proposed USN obtains values of the underwater ground displacement. The displacement values that are obtained are used in the planning phase while determining the suitable locations for hosting UDCs. In the planning phase, the USN also incorporates sensors that measure the temperature of the underwater environment. Therefore, the USN incorporates sensors that monitor the observed ground displacement and temperature in the underwater environment. The output of the planning phase is data showing the ground displacement and temperature at different time instants for varying zones and locations in the underwater environment.
2. Second, the research proposes a novel computing architecture considering the paucity of suitable underwater locations. The considered paucity arises due to the joint consideration of the underwater ground displacement and temperature in determining the number of suitable underwater locations for hosting UDCs. The novel computing architecture considers the variations of ground displacement, observed temperature and marine heat wave occurrence. The novel computing architecture incorporates a service provider independent computing architecture. In the proposed service provider computing architecture (SPCA) paradigm, future service providers do not launch own UDCs. Instead, the service providers utilize UCD within the context of a defined service level agreement (SLA). The dynamic SLA’s definition has been motivated to enable the support of service providers who do not have sufficient resources to develop and deploy own UDCs. In addition, the use of a dynamic SLA enables the maximal utilization of suitable ocean’s zones capable of hosting UDCs. Space limitation becomes a concern due to the consideration of the extra constraint and criterion of underwater ground displacement.
3. The presented research also formulates the performance metric. The performance metric is the operational duration. The operational duration is formulated before and after the use of the proposed mechanism. This is done to consider the influence of underwater seismic activity on the operational duration and capability of the UDC. In addition, a UDC with reservoir is considered in the performance modelling and simulation. The PUE of the UDC with a reservoir is formulated and examined in the context of the use of the proposed solution. The aim of the formulation and evaluation is to investigate how the consideration of seismic activity influences the achievable PUE when the UDC incorporates a reservoir.

The rest of the paper is organized as follows. Section “Existig work” focuses on background work. Section “Problem description” is the problem description. Section “Proposed solution” presents the proposed solution. The performance model is formulated in Section “Performance formulation”. Section “Performance evaluation” discusses the performance evaluation. Section “Conclusion” concludes the presented research.

## Existing work

The suitability of the ocean to host future computing platforms is recognized in^14^. The consideration in^14^ addresses the allocation of ocean location resources for UDC hosting. The use of the ocean location is recognized to motivate the design of a new computing architecture. The discussion in^14^ proposes the utilization of data center stacking within a multi-vendor context. The main pressure affecting ocean location resource allocation is the suitability of a location for cooling. The research in^14^ also proposes an intelligent solution to identify the suitable locations for UDC hosting. The occurrence of dynamic marine heat waves is recognized as an event influencing UDC operational duration in^15^.

The research in^15^ proposes the incorporation of a reservoir into the UDC. The reservoir stores water that is later used for UDC cooling when marine heatwaves occur. The research demonstrates that using UDC (with integrated reservoirs) enhances the UDC operational duration. The study in^15^ recognizes the ocean as a dynamic environment where different events influence free cooling water availability. In this case, the marine heat wave is a coolant availability limiting event. However, the framework presented in^15^ has not considered how to ensure UDC functioning given the occurrence of the ocean environment limiting events.

Dar et al.^19^ focuses on the application deployment of a UDC derivative, the underwater fog computing solution. The performance of the platforms such as the Raspberry Pi, and supporting hardware in realizing the underwater fog i.e., the micro-cloud data center i.e., the underwater fog computing system is examined. However, the role of sensors to demonstrate how the occurrence of ocean events that limit the availability of free cooling water affect the deployed underwater fog computing system requires additional consideration. The challenge of assessing the cooling performance and addressing the logistical challenges is considered in^20^. The logistical challenges being addressed is that of packing servers into UDCs. The packing solution considers the positioning of the cooling system, server cabinet configurations, and server power density. The focus of Hu et al.^20^ is on ensuring that the UDC interior does not impair the UDC performance. The subject has not addressed the effectiveness of the interior with relation to the occurrence of events in the surrounding ocean environment.

Sutherland et al.^21^ provide a review of Microsoft Project Natick, a pioneer UDC. The performance concerns that have been identified are the operational failure rate and harnessing renewable energy via the use of ocean currents in renewable energy. It is also recognized that the use of UDCs raises environment impact concerns. However, it is important to note that the uptime i.e., operational duration has not received consideration from the Microsoft Natick initiative considering the occurrence of other events in the ocean environment. The study in^21^ has focused on how to ensure that the deployment of UDC does not lead to the significant environmental degradation.

The use and adoption of UDC though at a nascent stage has necessitated the design of environment alert systems. Kadambi et al.^22^ propose an environment alert system for monitoring the health status of a pod based UDC. The sensors in^22^ are motion detectors, gas sensors, temperature sensors, ultrasonic distance sensors and piezo buzzers. These sensors are deployed to monitor the threats from the ocean environment that can influence the UDC performance. However, an additional layer and suite of sensors that enable UDC adaptability to ocean events like marine heat waves and ocean’s seismic related events are required. However, the incorporated sensor layers do not monitor the onset of the occurrence of the ocean related events such as marine heat wavers and underwater seismic related events. The larger context of the use of underwater sensor networks has been seen in the case of acquiring underwater related data as seen in^23–25^. Sensors can also be used to monitor for the occurrence of marine heat waves^26–28^, and volcanic underwater activity^29–31^.

The observations described in^26–28^ and in^29–31^ are satellite data driven and does not utilize physically deployed underwater sensors. These observations utilize satellite data for inferring marine heat waves and underwater volcano activity. The utilization of meteorological geostationary satellites is beneficial in this case. The benefit arises from the large-scale coverage of meteorological geostationary satellites. However, the integration of satellite-based data on marine heat wave and volcanic activity though beneficial is yet to receive research consideration. In addition, the manner of identified integration is yet to receive consideration in the context of UDCs. In the context of the UDC, the occurrence of underwater volcanic activity leads to an increase in ocean temperature^32–34^.

The studies in^35–37^ provide an improved understanding of how volcanic activity affects the performance of UDCs. The search by Hansen et al.^36^ recognizes that underwater volcanoes can be regarded as underwater super storms. In addition, the discussion in^35–37^ enables the realization that underwater volcano occurrence results in ocean warming.

The discussion in^38–42^ present and describe the recent discovery of an increasing number of underwater volcanoes. The presented findings in^38–42^ note the existence of 43,000 underwater volcanoes. The number of 43,000 underwater volcanoes is not an upper limit and more underwater volcanoes can be discovered. The detection of this high number of underwater volcanoes has been enabled via the analytics of the associated satellite related data. The existence of a large number of underwater volcanoes necessitates the design of a solution enabling continued UDC functionality when underwater volcanic events occur. In addition, the proposed solution shouldn’t incorporate the role of satellite connectivity and communications. The non-inclusion of satellite related applications in this case is motivated by the need to reduce operational costs.

The occurrence of underwater volcanic activity should also be considered due to the discovery of more seamounts by studies in submarine geology^43–48^. Existing UDC deployment such as attempts from Microsoft Project Natick^49^, Subsea Cloud^50^, and Highlander^51^ have not considered the implications of findings from the domain of submarine geology. However, it is important to consider these findings from underwater geology. For example, Subsea cloud has recognized that siting UDCs at greater underwater depths enhances their physical security. The enhancement of the physical security improves their cybersecurity appeal. However, this benefit gives rise to the challenge that being at a greater depth leads to increased exposure to ocean warming due to the activity of underwater volcanoes and seamounts related emissions.

The solution in^15^ proposes the use of reservoirs to ensure continued UDC functioning given that marine heat waves occur. The event of underwater volcanoes increases reservoir cooling load. The approach of using reservoirs becomes unscalable when more reservoirs are required. Increasing the number of reservoirs also results in an increased overhead. The use of more reservoirs leads to increased overhead due to the need to coordinate reservoir activation and de-activation. The increased overhead arises because of more focus on deploying more reservoirs (which begins to consume more resources) increasingly than the core computing functionality of the UDC.

The examined research shows that UDC use though nascent is receiving consideration. The use of the ocean environment for UDC hosting experiences challenges from marine heat waves. Marine heat waves increase the ocean temperature. Therefore, marine heat waves reduce the availability of cold water for UDC cooling. The use of reservoirs that store cold water to be used for later cooling has been proposed in ^15^. The use of reservoirs can be deemed suitable for ensuring continued UDC functionality when other events that leads to an increase in ocean temperature occurs. However, the use of reservoirs poses the challenge of increasing UDC operation overhead. It is important to address the challenge of reducing the UDC overhead.

Therefore, a proposed approach that addresses this challenge without incurring significant UDC associated overhead is required. The description of such a solution is the goal of the presented research.

## Problem description

The scenario comprises multiple UDCs located in the epipelagic and mesopelagic zones. The concerned UDCs do not benefit from information related to satellite systems and networks. In addition, the UDCs are connected to external networks via high-speed fibre optic cables. These external networks engage with the UDC via network gateway systems. Furthermore, the UDC utilizes a mix of renewable and non–renewable sources for operation. Each of the UDC hosts reservoir systems that enable cooling in the event of maritime heat waves. This is the case of the UDC model presented in^15^. The set of UDCs is denoted $\alpha$ such that:

1 $$\alpha =\left\{{\alpha }_{1},{\alpha }_{2},\dots , {\alpha }_{A}\right\}$$

A given reservoir can be associated with a set of several reservoirs. The set of reservoirs associated with the *a*th reservoir, ${\alpha }_{a},{\alpha }_{a} \epsilon \alpha$ is given as:

2 $${\alpha }_{a}=\left\{{\alpha }_{a}^{1},{\alpha }_{a}^{2},\dots , {\alpha }_{a}^{B}\right\}$$

Let ${I}_{F}\left({\alpha }_{a},{t}_{y}\right), {t}_{y} \epsilon t, t=\{{t}_{1},\dots ,\boldsymbol{ }{t}_{Y}\}$ be the functional status of the associated reservoir ${\alpha }_{a}$ at the epoch ${t}_{y}$. The duration of the marine heat wave associated with ${\alpha }_{a}$ is denoted ${\beta }_{1}\left({\alpha }_{a}\right)$. The computing workload associated with the reservoirs of ${\alpha }_{a}$ at the epoch ${t}_{y}$ is denoted as ${C}_{1}\left({\alpha }_{a}^{b} , {t}_{y}\right), {\alpha }_{a}^{b} \epsilon {\alpha }_{a}.$ The expected functional duration of the UDC ${\alpha }_{a}$ is denoted ${\beta }_{exp}\left({\alpha }_{a}\right)$. In addition, the functional duration of the ${b}^{th}$ reservoir of the ${a}^{th}$ UDC, ${\alpha }_{a}^{b}$ is denoted as ${\beta }_{1}\left({\alpha }_{a}^{b}\right)$. The operation of the UDC to ensure continued functionality becomes increasingly challenging when:

3 $$\sum_{b=1}^{B}{\beta }_{1}\left({\alpha }_{a}^{b}\right)< {\beta }_{exp}\left({\alpha }_{a}\right)$$

4 $$\sum_{b=1}^{B}\sum_{y=1}^{Y}\left({C}_{1}\left({\alpha }_{a}^{b} , {t}_{y}\right)+ \theta \right) < {C}_{1}\left({\alpha }_{a}\right)$$

where ${C}_{1}\left({\alpha }_{a}\right)$ is the total computing resources i.e., server capacity aboard the ${a}^{th}$ UDC, ${\alpha }_{a}$.

The scenario modelled by (3) describes the case when the total operational duration enabled by the reservoirs associated with ${\alpha }_{a}$ is less than the expected operational duration of the UDC ${\alpha }_{a}$.

In (4), the term $\theta$ describes the computing resources utilized by the UDC ${\alpha }_{a}$ in ensuring continued UDC functionality when other underwater events besides marine heat waves occur. The utilization of computing resources in this execution of the reservoir monitoring process and enabling configuration leads to overhead. The overhead is the data arising due to the necessity of hosting multiple reservoirs and coordinating their functionality via supporting computing architectures. The overhead describes the amount of data being handled by the UDC which is not related to the process of executing the storage and processing of user data. A challenge of UDC reservoir increasing overhead arises when:

5 $$\left(\frac{1}{{C}_{1}\left({\alpha }_{a}\right)}\sum_{y=1}^{A}\sum_{b=1}^{B}{C}_{1}\left({\alpha }_{a}^{b} , {t}_{y}\right)\right)>\left(\frac{1}{{C}_{1}\left({\alpha }_{a}\right)}\sum_{y=A+1}^{x}\sum_{b=1}^{B}{C}_{1}\left({\alpha }_{a}^{b} , {t}_{y}\right) \right)> \left(\frac{1}{{C}_{1}\left({\alpha }_{a}\right)}\sum_{y=A+1}^{x}\sum_{b=1}^{B}{C}_{1}\left({\alpha }_{a}^{b} , {t}_{y}\right)\right)$$

## Proposed solution

The discussion here presents the proposed solution i.e., UDC with the ability to maintain continued functionality given the occurrence of underwater volcanoes. It is sub-divided into three aspects. The first focuses on the proposed underwater sensor network architecture. The second aspect discusses on the decision-making algorithm used by the proposed UDC. The third aspect presents the architecture of the proposed UDC.

### First aspect—underwater sensor network architecture

The underwater sensor network (USNA) is designed on the basis that underwater ground displacement profile is a precursor to underwater volcanic activity. The view in^52–54^ supports this notion. In the USNA, underwater ground displacement monitoring entities are deployed in different ocean regions. The underwater ground displacement monitoring entity (UDME) are sensors that are hosted aboard underwater autonomous underwater vehicles (AUVs). The concerned AUVs host UDMEs, and sensors that measure the temperature of different underwater locations. The proposed USNA comprise multiple AUVs that are able to exchange the data on underwater ground displacement and temperature with each other. Advances in underwater communications enable the realization of wireless AUV communications as seen in^55–58^.

The AUVs merge and exchange data with each other. The exchange is done alongside the execution of a relay to the UDC. Let $\phi$ denote the set of AUVs such that:

6 $$\phi =\left\{{\phi }_{1},{\phi }_{2},\dots , {\phi }_{J}\right\}$$

**Figure 1:**
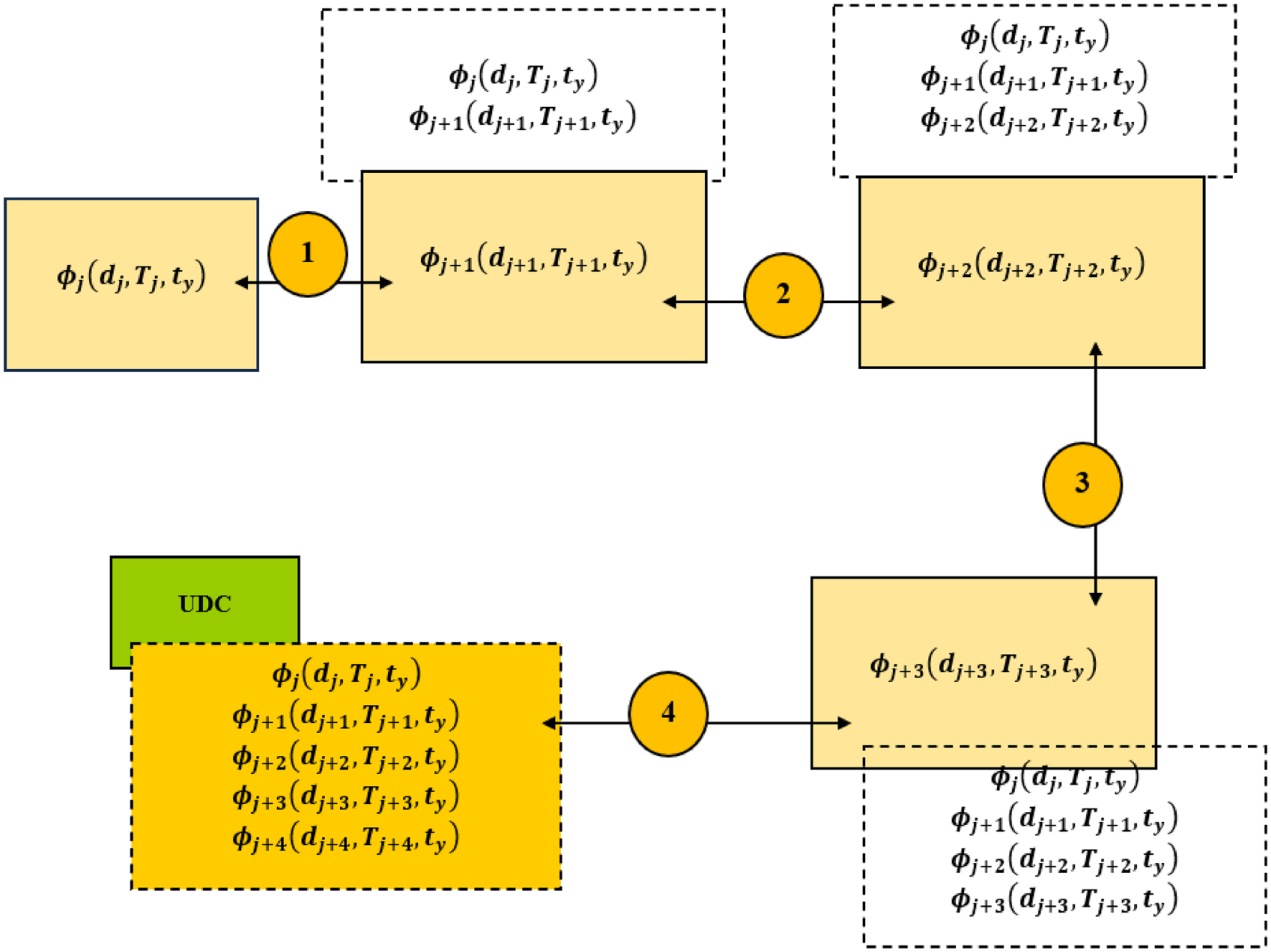
Data forwarding between underwater AUVs.

The data associated with the *j*th AUV, ${\phi }_{j} , {\phi }_{j} \epsilon \phi$ at the epoch ${t}_{y}$ is denoted ${\phi }_{j}\left({d}_{j},{T}_{j},{t}_{y}\right)$. In ${\phi }_{j}\left({d}_{j},{T}_{j},{t}_{y}\right), {d}_{j}$, and ${T}_{j}$ signify the ground displacement and temperature associated with the AUV ${\phi }_{j}$ at the epoch ${t}_{y}$, respectively. Given that a network of AUVs ${\phi }_{j+1}, {\phi }_{j+1}\epsilon \phi , {\phi }_{j+2}, {\phi }_{j+2 }\epsilon \phi$ and ${\phi }_{j+3}, {\phi }_{j+3 }\epsilon \phi$ are linked. In this case, the AUVs ${\phi }_{j+3}$ engages in direct communications with the UDC. The data associated with ${\phi }_{j+1}, {\phi }_{j+2,}$ and ${\phi }_{j+3}$ at the epoch ${t}_{y}$ are ${\phi }_{j+1}\left({d}_{j+1},{T}_{j+1},{t}_{y}\right), {\phi }_{j+2}\left({d}_{j+2},{T}_{j+2},{t}_{y}\right),$ and ${\phi }_{j+3}\left({d}_{j+3},{T}_{j+3},{t}_{y}\right),$ respectively. The manner of data forwarding between AUVs ${\phi }_{j},{\phi }_{j+1}, {\phi }_{j+2,}$ and ${\phi }_{j+3}$ and the UDC is presented in the Scenario described in Fig. 1.

The scenario in Fig. 1 shows the execution of data transfer between AUVs via acoustic communications. The distance between AUVs does not exceed the permissible acoustic transmission range. In the case of Fig. 1, the optical transmission achieves high data rate. This is realizable in the order of up to tens of megabits per second^59^. The discussion by Aman et al.^59^ notes that the acoustic modem can achieve data rates in the range (1.5–50) kbps to (0.6–3) kbps for 500 m, and (28–120) km, respectively. The use of acoustic modem is preferred. The rationale is the non–necessity of realizing a line of sight in comparison to optical underwater wireless communication networks. In addition, advances have been made in underwater sensor network architecture^60^.

Furthermore, the AUVs are battery powered. In the implied underwater sensor network, the sensors are hosted aboard the AUV. The data acquired by the sensors is stored by the AUV and relayed through the network. In the proposed network, the AUVs (and the borne sensors) sojourn to different ocean regions. Since, the AUVs and the borne sensors are mobile (capable of traversing different ocean depths and heights), the concerned network is a three dimensional underwater wireless sensor network.

**Figure 2:**
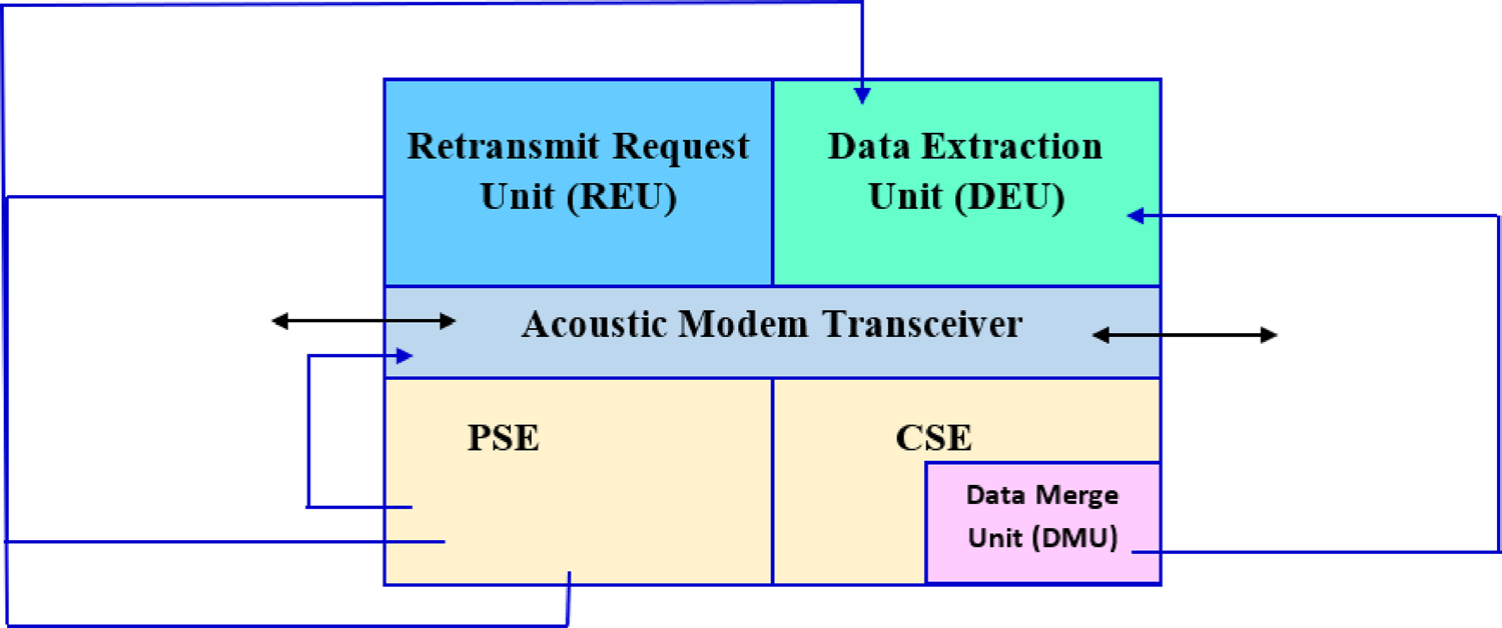
Architecture of the proposed AUV.

Each of the AUV presented in Fig. 1 comprise a computing subsystem entity (CSE), network subsystem entity (NSE), and power subsystem entity (PSE). The CSE executes tasks associated with merging data received from other AUVs i.e., neighbouring AUVs. The NSE hosts the acoustic modem (and transceiver) and engages in data exchange with neighbouring AUVs. In addition, the PSE hosts the battery and components enabling the powering of the CSE, and the NSE. The architecture of the proposed AUV is shown in Fig. 2.

Figure 2 shows the AUV architecture, the presented AUV hosts the request retransmit unit (RRU), and the data extraction unit (DEU). The RRU enables the NSE to request for data that has not been delivered. The DEU enables the receipt of data from the transceiver, and access to merged data from the data merge unit (DMU) hosted on the CSE.

### Second aspect—decision making

The decision-making process using the obtained data is executed aboard the UDC. This is executed on the data analytics and decision-making entity (DME). For the purpose of the discussion, the ground displacement and temperature are considered as separate parameters. Therefore, the parameter ${\phi }_{j}\left({d}_{j},{T}_{j},{t}_{y}\right)$ is re-written as ${\phi }_{j}\left({d}_{j},{t}_{y}\right),$ and ${\phi }_{j}\left({T}_{j},{t}_{y}\right)$. The DME receives parameters for multiple AUVs. An ocean location corresponding to the AUV, ${\phi }_{j}$ is deemed unsuitable when:

7 $${\phi }_{j}\left(x,{t}_{y}\right)< {\phi }_{j}\left(x,{t}_{y+1}\right)< {\phi }_{j}\left(x,{t}_{y+2}\right)<\dots .<{\phi }_{j}\left(x,{t}_{y+p}\right), {t}_{y+1}\epsilon {t}_{y}, {t}_{y+2} \epsilon {t}_{y}, {t}_{y+p}\epsilon {t}_{y}$$

8 $$x \epsilon \left\{{d}_{j}, {T}_{j}\right\}$$

9 $$\frac{1}{(A-1)}\sum_{y=1}^{A}{\phi }_{j}\left(x,{t}_{y}\right)<\frac{1}{(B-A-2)}\sum_{y=A+1}^{B}{\phi }_{j}\left(x,{t}_{y}\right) < \frac{1}{(Y-B-2)}\sum_{y=B+1}^{Y}{\phi }_{j}\left(x,{t}_{y}\right)$$

The relations in (7)–(9) describe the case where the ocean region associated with the ${j}^{th}$ AUV, ${\phi }_{j}$ has increasing ground displacement. In this case, the increasing ground displacement is accompanied with a rising temperature in the ocean region. The observation underlying the model presented in (7)–(9) occurs over a given duration. The concerned duration spans the start epochs starting at ${t}_{y+1},$ and end epoch ${t}_{y+p}$. The scenario described by the relation (7)–(9) is called Profile 1 of the underwater environment. The ocean location is also deemed unsuitable when:

10 $${\phi }_{j}\left(x,{t}_{y}\right)< {\phi }_{j}\left(x,{t}_{y+1}\right)> {\phi }_{j}\left(x,{t}_{y+2}\right)<\dots <{\phi }_{j}\left(x,{t}_{y+p}\right), {t}_{y+1}\epsilon {t}_{y}, {t}_{y+2} \epsilon {t}_{y}, {t}_{y+p}\epsilon {t}_{y}$$

11 $$x \epsilon \left\{{d}_{j}, {T}_{j}\right\}$$

12 $$\frac{1}{(A-1)}\sum_{y=1}^{A}{\phi }_{j}\left(x,{t}_{y}\right)<\frac{1}{(B-A-2)}\sum_{y=A+1}^{B}{\phi }_{j}\left(x,{t}_{y}\right)> \frac{1}{(Y-B-2)}\sum_{y=B+1}^{Y}{\phi }_{j}\left(x,{t}_{y}\right)$$

The relations in (10)–(12) describe a case where there is a momentary reduction in the underwater ground displacement and ocean temperature. The AUV being considered is the *j*th AUV ${\phi }_{j}$ for the same epochs as those considered in (7)–(9). The scenario described in (10)–(12) is considered as Profile 2.

Besides Profile 1, and Profile 2 another plausible case is one in which the concerned ocean region is deemed suitable. This case is called Profile 3 and described as:

13 $${\phi }_{j}\left(x,{t}_{y}\right)> {\phi }_{j}\left(x,{t}_{y+1}\right)> {\phi }_{j}\left(x,{t}_{y+2}\right)>\dots >{\phi }_{j}\left(x,{t}_{y+p}\right), {t}_{y+1}\epsilon {t}_{y}, {t}_{y+2} \epsilon {t}_{y}, {t}_{y+p}\epsilon {t}_{y}$$

14 $$x \epsilon \left\{{d}_{j}, {T}_{j}\right\}$$

15 $$\frac{1}{(A-1)}\sum_{y=1}^{A}{\phi }_{j}\left(x,{t}_{y}\right)>\frac{1}{(B-A-2)}\sum_{y=A+1}^{B}{\phi }_{j}\left(x,{t}_{y}\right)> \frac{1}{(Y-B-2)}\sum_{y=B+1}^{Y}{\phi }_{j}\left(x,{t}_{y}\right)$$

The case of Profile 3 described by the relations in (13)–(15) presents a case where the ocean region is settling from a ground displacement increase and temperature increase i.e., warming related disturbances. An observation showing the validity of the relations in (13)–(15) indicates the suitability of the ocean location when a long duration is concerned. In addition, another profile i.e., Profile 4 indicates a stable ocean region. Profile 4 is described as:

16 $${\phi }_{j}\left(x,{t}_{y}\right)\approx {\phi }_{j}\left(x,{t}_{y+1}\right)\approx {\phi }_{j}\left(x,{t}_{y+2}\right)\approx \dots .\approx \approx {\phi }_{j}\left(x,{t}_{y+p}\right), {t}_{y+1}\epsilon {t}_{y}, {t}_{y+2} \epsilon {t}_{y}, {t}_{y+p}\epsilon {t}_{y}$$

17 $$x \epsilon \left\{{d}_{j}, {T}_{j}\right\}$$

18 $$\frac{1}{(A-1)}\sum_{y=1}^{A}{\phi }_{j}\left(x,{t}_{y}\right)\approx \frac{1}{(B-A-2)}\sum_{y=A+1}^{B}{\phi }_{j}\left(x,{t}_{y}\right)\approx \frac{1}{(Y-B-2)}\sum_{y=B+1}^{Y}{\phi }_{j}\left(x,{t}_{y}\right)$$

### Third aspect—proposed UDC architecture

**Figure 3:**
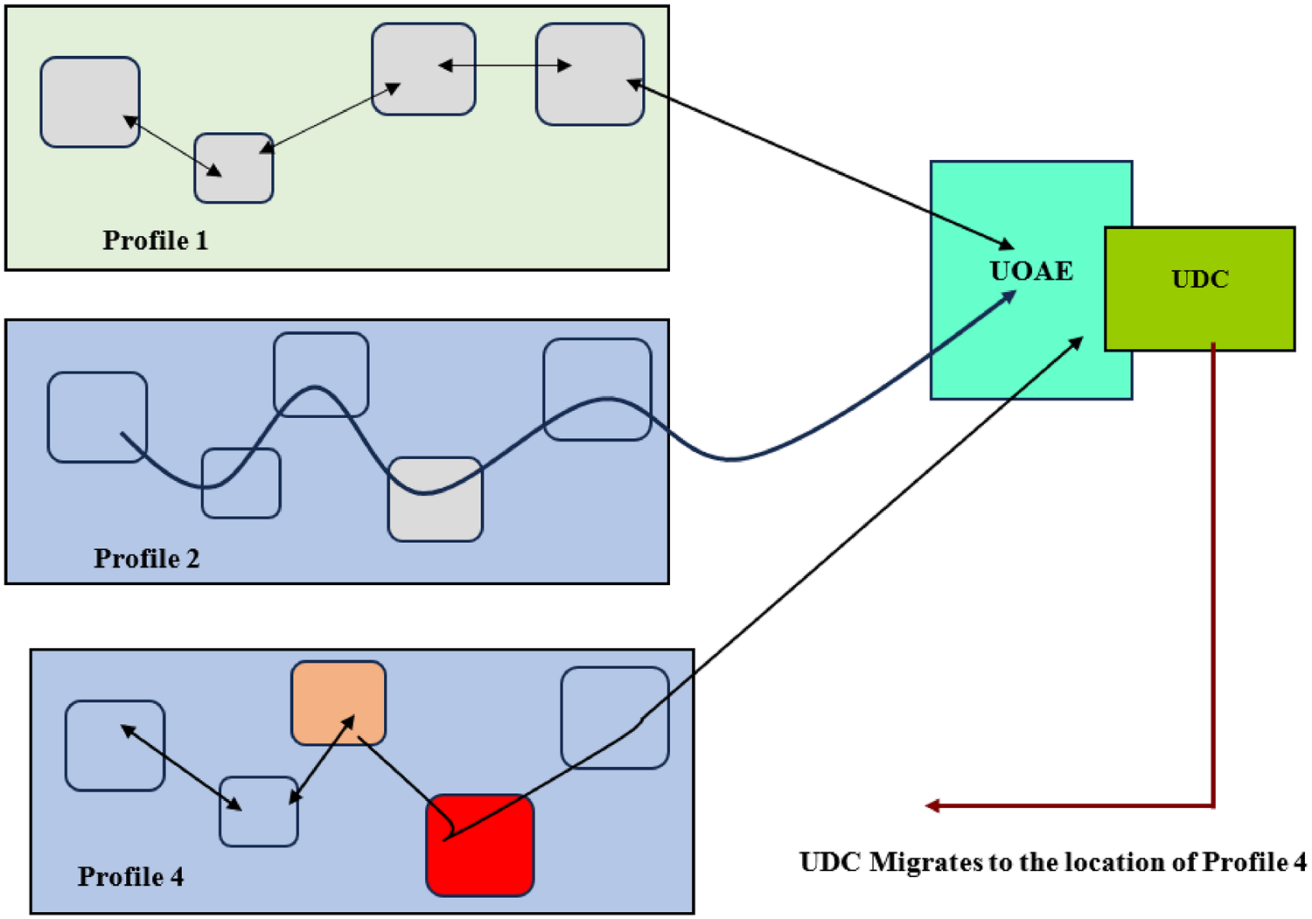
Role of the UOAE in the proposed UDC and system.

The proposed UDC is presented and discussed in this aspect. The UDC infers information on each different ocean region’s profile and status. The inference is done using information on the underwater ground displacement and temperature. The UDC executed the functionality of classifying the ocean region using the data received from the acoustic modem transceiver aboard the AUV. The classification is done by the UDC open analytic entity (UOAE). In the proposed UDC, the design approach does not focus on incorporating more reservoirs as found in^15^. In addition, the proposed approach incorporates the capability of mobility in the UDC. The proposed UDC is able to execute manoeuvres enabling a change in its location. Manoeuvrability in the proposed UDC is challenging due to the inability of the geographical positioning system to function underwater. The interaction between the AUV network and the UDC is used to realize this expected capability expected. Each AUV at a given hop appends its identifier to the merged data prior to forwarding. The identifier is appended alongside the distance between the communicating AUVs. Each forwarding path is associated with the set of AUVs constituting the different hops. The information on each forwarding path and AUV identifiers are used by the UDC’s UOAE in region-based profile classification. The profile classification is done to determine the profile associated with a given AUV path. The execution of a UDC manoeuvre to migrate to an ocean region classified as Profile 3 is in Fig. 3.

Figure 3 shows an ocean region with the profiles Profile 1, Profile 2, and Profile 4. The presentation in Fig. 3 shows each of the profile associated hop has a connection to the UOAE. The UOAE executes the relations presented in (7)–(18). An identification of a zone as being classified Profile 4 or other most suitable profile is followed by a sojourn manoeuvre. The sojourn manoeuvre is preceded by path confirmation execution (PCE). In the PCE, the UDC sends out information on the AUV identifiers on the path associated with the data corresponding to the best profile. Each AUV whose identifies is in this information responds to the UDC as being present. A successful receipt of this information is followed by the execution of the UDC manoeuvre sojourn. The proposed UDC has an extensible length of high-speed optic fibre cable to realize connection to external network gateways.

The proposed UDC incorporates the service provider computing architecture (SPCA). In the SPCA, the UDC is not necessarily an asset of the service provider. For example, in the existing approach; UDCs that are deployed in the project Natick initiative are exclusively owned by Microsoft. The newly introduced SPCA enables Microsoft to run platforms similar to Project Natick without executing all tasks related to harnessing the ocean’s resources. In the proposed SPCA, the utilization of UDCs is influenced by a service level agreement (SLA). The definition of the SLA comprises the (1) Identity of the requesting entity, (2) Minimum amount of computing resources, (3) Maximum amount of computing resources, and (4) Requestor duration. The requestor generates an SLA which is sent to the UDC operator. The required transmission is done over the internet as an exchange between the requestor gateway, and the operator gateway.

**Figure 4:**
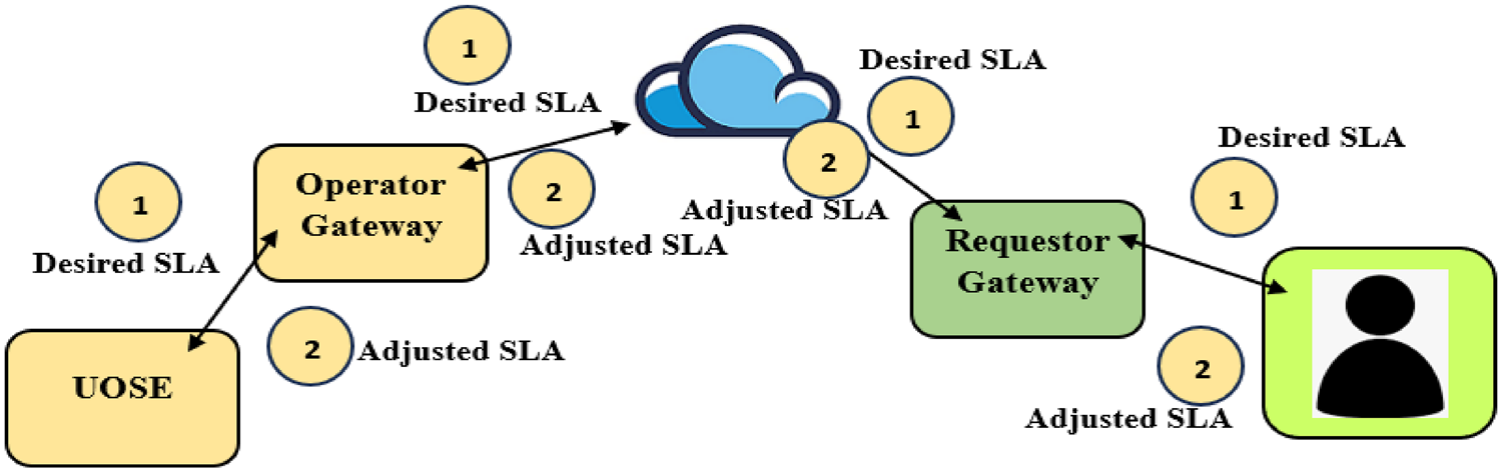
Scenario showing information exchange.

The operator gateway provides the received information to UDC operational status entity (UOSE). The UOSE evaluates the feasibility of granting the SLA from the requestor. In the event that a newly received SLA conflict with an existing SLA, the UOSE determines the SLA conditions that can be granted by the operator. The operator sends this information to the requestor via the requestor gateway–operator gateway internet driven exchange. The interchange being described is shown in Fig. 4.

**Figure 5:**
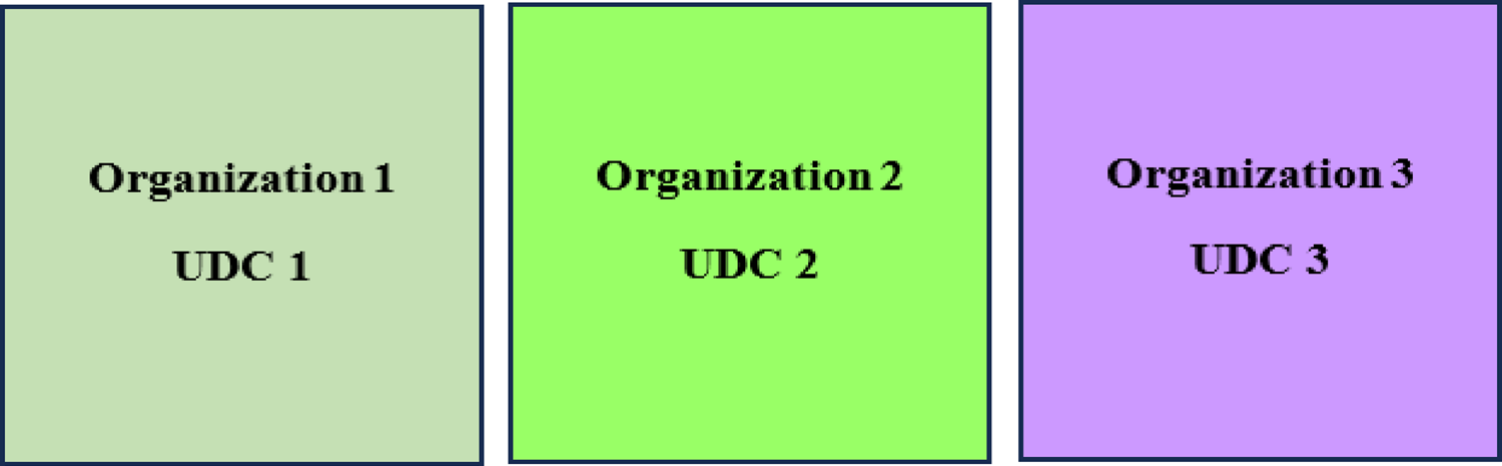
Manner of utilization of UDC by the owner organization in the existing approach.

The role of the SPCA as regards computing resource utilization in the existing and proposed solution is shown in Fig. 5. In Fig. 5, the scenario indicates the utilization of the UDC by a single organization i.e., the owner organization. The existing case is one in which each of the UDCs is owned and utilized by the owner organization. This is the existing case.

In Fig. 5, the concerned organizations i.e., organization 1, organization 2, and organization 3 are the owner organizations that exclusively use UDC 1, UDC 2, and UDC 3, respectively. They maintain a right to make use of the UDC’s server resources and do not share with each other. The approach is sub-optimal when owner organization workload does not require all time access and use of the UDC’s server computing resources. In this case, the drawback arising is that the UDC is being maintained in the underwater environment when servers are not utilized or are experiencing significant under–utilization.

**Figure 6:**
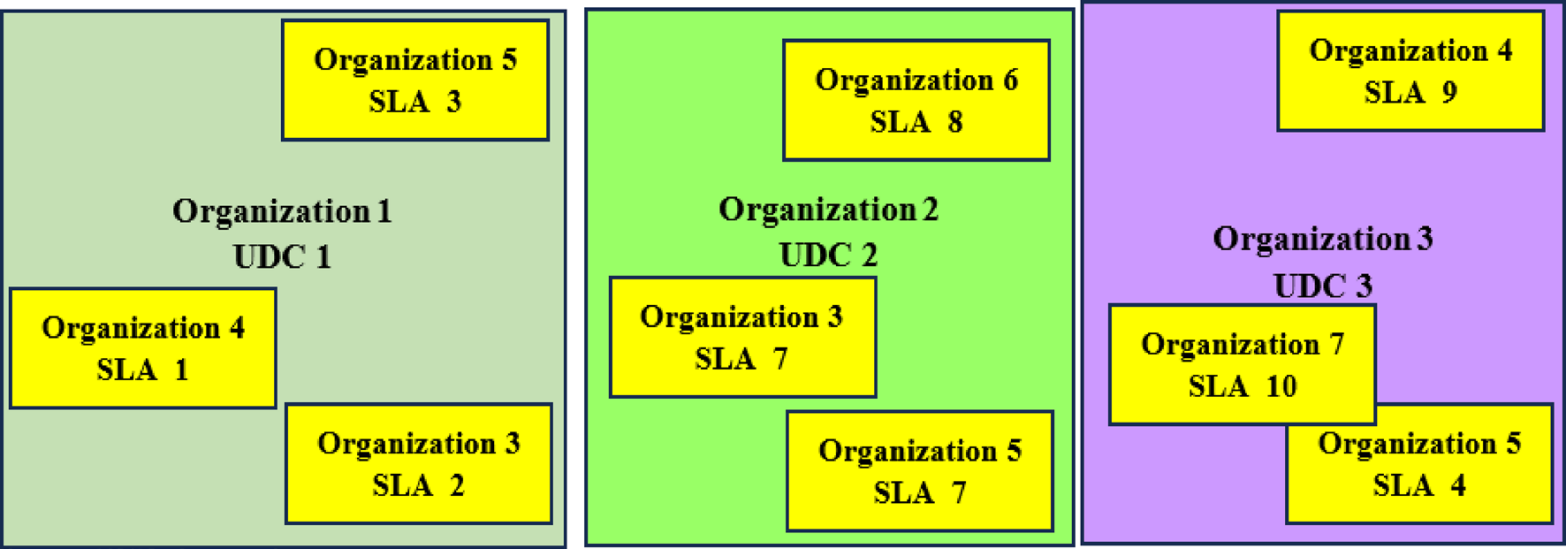
Scenario showing UDC utilization by multiple organizations incorporating the SPCA.

The proposed case incorporating the use of the SPCA algorithm is presented in Fig. 6. In this case, UDC 1 owned and operated by Organization 1 is utilized by Organization 3, Organization 4, and Organization 5 having SLAs, SLA 2, SLA 1, and SLA 3, respectively. In this case, the owner organization i.e., Organization 1 has a low or non–existent computing workload. The allocation of the idle computing resources to organizations 3, 4, and 5 in this case improves computing resource utilization. In addition, the allocation of idle computing resources presents a potential revenue source for the organization i.e., organization 1. Furthermore, the UDC 2 owned and operated by Organization 2 is utilized by Organization 3, Organization 5, and Organization 6 having SLAs, SLA 7, SLA 5, and SLA 8, respectively. In addition, the UDC 3 owned and operated by Organization 3 is utilized by Organization 4, Organization 5, and Organization 7 having SLAs, SLA 9, SLA 4, and SLA 10, respectively. The sharing of the resources aboard UDC 2, and UDC 3 enables the prevention of the occurrence of computing resource utilization for organization 2, and organization 3, respectively. The benefit is derived in a manner similar to associated with organization 1. Furthermore, the organization 2 and organization 3 host the computing demands for other applications which present a potential revenue source. The architecture in Fig. 6 is feasible. The feasibility is due to the increasing role of data in underwater technologies and applications^61–63^.

The scenario in Fig. 6 shows the case where the UDC owner organizations each experiencing idling server computing resources support three other organizations. The choice of three organizations is not intended to be restrictive. Each of the three-owner organization owning a physical UDC infrastructure can host more supporting organizations. In addition, the three UDCs in Fig. 6 i.e., UDC 1, UDC 2, and UDC 3 are in a region that is not affected by the occurrence of underwater volcanic activity.

## Performance formulation

The discussion here formulates the performance metrics of the: (1) Operational Duration, and (2) Associated System Overhead. The metric of the operational duration is formulated for the existing case of the reservoir driven UDC. It is also formulated for the case of the proposed UDC with incorporation of the mobility feature. The associated system overhead is formulated for the case of the existing reservoir driven UDC, UDC with incorporated mobility (proposed case), and UDC with incorporated mobility with SPCA. In the existing approach^15^, the metric of the operational duration ${\theta }_{1}$ is given as:

19 $${\theta }_{1}= \sum_{a=1}^{A}\sum_{b=1}^{B}{\beta }_{1}\left({\alpha }_{a}^{b}\right)$$

In the proposed approach, the metric of the operational duration, ${\theta }_{2}$ is given as:

20 $$ \begin{gathered} \theta_{2} = \mathop \sum \limits_{a = 1}^{A} \mathop \sum \limits_{b = 1}^{B} \beta_{1} \left( {\alpha_{a}^{b} } \right)\left( {1 - \vartheta_{1} \left( {\alpha_{a}^{b} } \right)} \right) + \mathop \sum \limits_{j = 1}^{J} \mathop \sum \limits_{y = 1}^{Y} B_{1} \left( {d_{j} ,T_{j} ,t_{y} } \right)\left( {1 - \vartheta_{2} \left( {d_{j} ,T_{j} ,t_{y} } \right)} \right) \hfill \\ \vartheta_{1} \left( {\alpha_{a}^{b} } \right) \le 1, 0 \le \vartheta_{2} \left( {d_{j} ,T_{j} ,t_{y} } \right) \le 1 \hfill \\ \end{gathered} $$

${\vartheta }_{1}\left({\alpha }_{a}^{b}\right)$ is the proportion of reservoir functioning duration during which the *b*th reservoir on the *a*th UDC provides cooling prior to the initiation of UDC migration to another region with improved profile. ${B}_{1}({d}_{j},{T}_{j},{t}_{y})$ is the duration associated with the ocean region hosting the *j*th AUV that observes the data on underwater ground displacement ${d}_{j}$, underwater temperature ${T}_{j}$ at the epoch ${t}_{y}$. ${\vartheta }_{2}\left({d}_{j},{T}_{j},{t}_{y}\right)$ is the proportion of the duration that the UDC spends in the location i.e., ocean region of the *j*th AUV.

The overhead is also formulated as an important metric and describes the proportion of computing resources used for other tasks besides that of conventional data storage and processing. The overhead in the existing approach i.e.,^15^, is denoted ${\theta }_{1}^{\prime}$, associated with the *a*th UDC is given as:

21 $${\theta }_{1}^{\prime}= \sum_{y=1}^{Y}\sum_{b=1}^{B}\frac{{C}_{1}\left({\alpha }_{a}^{b},{t}_{y}\right)+ {C}_{2}\left({\alpha }_{a}^{b},{t}_{y}\right) }{{C}_{1}({\alpha }_{a})}$$

${C}_{2}\left({\alpha }_{a}^{b},{t}_{y}\right)$ is the computing resources used by the ${b}^{th}$ reservoir on the ${a}^{th}$ UDC to ensure continual functioning when events like underwater seismic related activity occur. The computing resources in this case is associated with the incorporation of additional reservoirs to ensure continued functionality when the underwater seismic related events occur.

The relations in (21) raises the notion of reservoir scalability for two types of reservoirs. The first set of reservoirs and second set of reservoirs are incorporated into the UDC to enable functioning when marine heat waves, and underwater volcanic activity occur, respectively. Additional reservoirs can be included to enable cold water storage for UDC cooling. The additional reservoirs are included considering the occurrence of other ocean related events that cause ocean warming. The computing resources utilized by the set of reservoirs is denoted ${C}_{z}\left({\alpha }_{a}^{b},{t}_{y}\right)$. In addition, the overhead in this case ${\theta }_{1}^{{\prime}{\prime}}$ associated with the *a*th UDC is:

22 $${\theta }_{1}^{{\prime}{\prime}}= \sum_{y=1}^{Y}\sum_{b=1}^{B}\frac{{C}_{1}\left({\alpha }_{a}^{b},{t}_{y}\right)+ {C}_{2}\left({\alpha }_{a}^{b},{t}_{y}\right)+{C}_{z}\left({\alpha }_{a}^{b},{t}_{y}\right) }{{C}_{1}({\alpha }_{a})}$$

The overhead considering that multiple reservoir groups can be added to the UDC to enable continued functionality when different ocean warming related events occur is formulated. The concerned overhead in this case is denoted ${\theta }_{1}^{sca}$ and given as:

23 $${\theta }_{1}^{sca}= \sum_{y=1}^{Y}\sum_{b=1}^{B}\sum_{c=1}^{C}\sum_{d=1}^{D}\frac{{C}_{1}\left({\alpha }_{a}^{b},{t}_{y}\right)+ {C}_{2}\left({\alpha }_{a}^{c},{t}_{y}\right)+{C}_{z}\left({\alpha }_{a}^{d},{t}_{y}\right)}{{C}_{1}({\alpha }_{a})}$$

**Figure 7:**
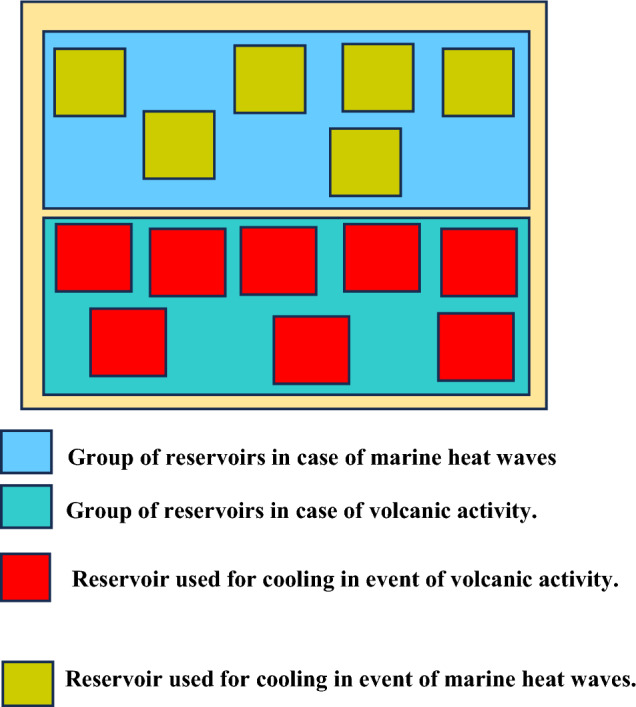
Reservoir architecture in existing case.

**Figure 8:**
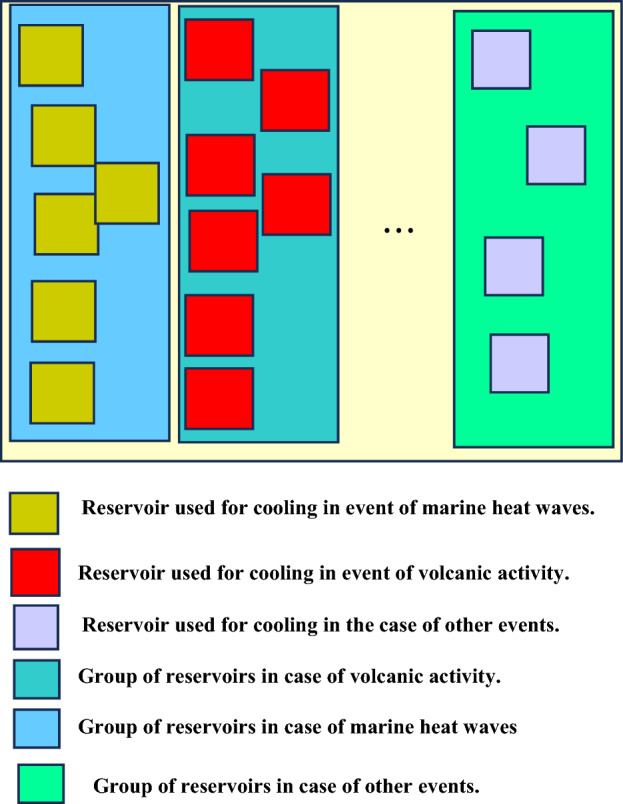
Reservoir architecture in proposed case.

The overhead in (22) considers that the UDC utilizes a set of reservoirs to provide continued UDC functioning when ocean warming occurs due to marine heat waves, and underwater volcanic activity. Another feasible scenario is one in which the UDC hosts multiple reservoir types. The inclusion of multiple reservoirs ensures continued UDC functionality. Such an approach accommodates the occurrence of different events that can lead to an increase in the ocean temperature. The case of the scenario in (22) and (23) are shown the scenarios described in Figs. 7, and 8, respectively.

In (23), the number of reservoirs used to store cold water for continued UDC functionality given the occurrence of Marine Heat Waves, Seismic related activity, and other ocean warming related events are $B, C,$ and $D,$ respectively. The relations in (23) incorporates reservoir heterogeneity i.e., the reservoir have different capabilities. Furthermore, it is feasible to have the concerned UDC communicate with a reservoir tower i.e., an external reservoir service provided by an underwater service provider. In this case, the communications is enabled via the use of AUVs.

Let the amount of computing resources for a case of $m$ paths with up to $n$ AUVs per path at the epoch ${t}_{y}$ be denoted as ${C}_{1}\left( {\phi }_{j}^{m},\dots , {\phi }_{j+n}^{m}, {t}_{y}\right), m=\left\{\mathrm{1,2},\dots , M\right\}$. The overhead considering the role of the AUVs is denoted ${\theta }_{1,tow}^{sca}$ and is given as:

24 $${\theta }_{1,tow}^{sca}= \sum_{y=1}^{Y}\sum_{b=1}^{B}\sum_{c=1}^{C}\sum_{d=1}^{D}\sum_{m=1}^{M}\sum_{n=1}^{N}\frac{{C}_{1}\left({\alpha }_{a}^{b},{t}_{y}\right)+ {C}_{2}\left({\alpha }_{a}^{c},{t}_{y}\right)+{C}_{z}\left({\alpha }_{a}^{d},{t}_{y}\right)+{C}_{1}\left( {\phi }_{j}^{m},\dots , {\phi }_{j+n}^{m}, {t}_{y}\right)}{{C}_{1}({\alpha }_{a})}$$

However, this approach has not been considered here because of the increased total ownership costs. The total ownership costs arise due to the necessity of the role of AUVs for providing communications, determining the volume of water required and accessing the required volume of water.

In the proposed approach, the UDC processes data from the AUVs along the paths having different profiles. The considered profiles are Profile 1, Profile 2, Profile 3, and Profile 4. The computing resources associated with each of the profiles is influenced by the number of corresponding AUV paths. Let the amount of computing resources for a case of $m$ paths with up to $n$ AUVs per path at the epoch ${t}_{y}$ for the profile $p$ be denoted as ${C}_{p}\left( {\phi }_{j}^{m},\dots , {\phi }_{j+n}^{m}, {t}_{y}\right), m=\left\{\mathrm{1,2},\dots , M\right\}$. The overhead in the proposed approach, ${\theta }_{2}$ considering that the migration results in the increased use of computing resources by $\varsigma_{p} \left( {C_{p} \left( { \phi_{j}^{m} , \ldots , \phi_{j + n}^{m} , t_{y} } \right)} \right), 0 \le \varsigma_{p} \left( {C_{p} \left( { \phi_{j}^{m} , \ldots , \phi_{j + n}^{m} , t_{y} } \right)} \right) \le 1$ is given as:

25 $$ \theta_{2} = { }\mathop \sum \limits_{y = 1}^{Y} \mathop \sum \limits_{m = 1}^{M} \mathop \sum \limits_{j = 1}^{J} \mathop \sum \limits_{n = 1}^{N} { }\frac{{C_{p} \left( {{ }\phi_{j}^{m} , \ldots ,{ }\phi_{j + n}^{m} ,{ }t_{y} } \right)\left( {1 + \varsigma_{p} \left( {C_{p} \left( {{ }\phi_{j}^{m} , \ldots ,{ }\phi_{j + n}^{m} ,{ }t_{y} } \right)} \right)} \right)}}{{C_{1} \left( {\alpha_{a} } \right)}}{ },{ }p\;\epsilon { }\left\{ {1,2,3,4} \right\} $$

The performance usage effectiveness (PUE) is formulated for the UDC. The power consumption in the UDC is divided into three aspects. These three aspects are power consumption by the (1) server and networking payload, (2) heat exchange payload, and (3) reservoirs. Let ${P}_{s}\left({\alpha }_{a},{t}_{y}\right),$ and ${P}_{C}\left({\alpha }_{a},{t}_{y}\right),$ denote the operational and cooling power for the servers aboard the *a*th UDC ${\alpha }_{a}$ at the *y*th epoch ${t}_{y}$, respectively. The power expended in operating the reservoir associated with *b*th reservoir of the *a*th UDC at the epoch ${t}_{y}$ is denoted ${P}_{1}\left({\alpha }_{a}^{b},{t}_{y}\right)$. In the existing case (with incorporation of the reservoir but with no mobility support) as seen in^15^, the PUE for the ${a}^{th}$ UDC ${\alpha }_{a}$ is denoted ${\Gamma }_{1}$ and given as:

26 $${\Gamma }_{1}= \sum_{y=1}^{Y}\sum_{b=1}^{B}\left(\frac{{P}_{s}\left({\alpha }_{a},{t}_{y}\right)+{P}_{C}\left({\alpha }_{a},{t}_{y}\right)\left(1-{\gamma }_{C}\left({\alpha }_{a},{t}_{y}\right)\right)+{P}_{1}\left({\alpha }_{a}^{b},{t}_{y}\right)\left(1+{\psi }_{1}\left({\alpha }_{a}^{b},{t}_{y}\right)\right) }{{P}_{s}\left({\alpha }_{a},{t}_{y}\right)}\right)$$

${\gamma }_{C}\left({\alpha }_{a},{t}_{y}\right)$ is the reduction in the cooling server due to the low temperature environment of the ocean hosting the UDC. In (26), the value of ${\gamma }_{C}\left({\alpha }_{a},{t}_{y}\right)$ is described as $0\le {\gamma }_{C}\left({\alpha }_{a},{t}_{y}\right)\le 1$. ${\psi }_{1}\left({\alpha }_{a}^{b},{t}_{y}\right)$ is the percentage increase in the power consumption of reservoir associated operation. It arises due to the need to consider continued UDC functionality due to the occurrences of several events that can lead to ocean warming. The value of ${\psi }_{1}\left({\alpha }_{a}^{b},{t}_{y}\right)$ is described as $0\le {\psi }_{1}\left({\alpha }_{a}^{b},{t}_{y}\right)\le 1$.

In (26), the parameter ${P}_{C}\left({\alpha }_{a},{t}_{y}\right)$ is the power that would have been used in server cooling if the server were in a terrestrial environment i.e., conventional deployment.

The PUE in the case of the proposed approach (with mobility support and reducing influence of reservoir support) for the ${a}^{th}$ UDC ${\alpha }_{a}$ is denoted ${\Gamma }_{2}$ and given as:

27 $${\Gamma }_{2}= \sum_{y=1}^{Y}\sum_{b=1}^{B}\left(\frac{{P}_{s}\left({\alpha }_{a},{t}_{y}\right)+{P}_{C}\left({\alpha }_{a},{t}_{y}\right)\left(1-{\gamma }_{C}\left({\alpha }_{a},{t}_{y}\right)\right)+{P}_{1}\left({\alpha }_{a}^{b},{t}_{y}\right)\left(1-{\psi }_{1}\left({\alpha }_{a}^{b},{t}_{y}\right)\right) }{{P}_{s}\left({\alpha }_{a},{t}_{y}\right)}\right)$$

## Performance evaluation

The discussion here presents the performance evaluation for the concerned metrics of the operational duration, overhead, and the power usage effectiveness (PUE). The performance evaluation proceeds as follows. The first part presents the results on operational duration. The second part discusses results on the overhead. The third part focuses on the PUE.

### First aspect—evaluation and analysis of the operational duration

The parameters used for the MATLAB simulation in the case of the operational duration is presented in Table 1. In evaluating the performance benefit, the reservoir considering the scenario in the existing approach has significant technological improvement in comparison to the reservoir used in the proposed case. This approach has been considered for two reasons. In the first case, this helps the conduct of the performance analysis and simulation in a non-greedy manner. Secondly, it is done assuming that the UDC operator significantly deploys the best technology to enhance the reservoir capacity given that there are no extra reservoirs to ensure continued UDC functionality when ocean warming related events occur. The concerned parameter associated with the second consideration is that of the percentage improvement in the functionality of the existing reservoir. Three values have bene considered for the percentage improvement as presented in the simulation parameters shown in Table 1. These values are 25, 50, and 75%.

**Table 1:** Investigation of the operational duration—the Simulation Parameters.

S/N	Parameter	Value
First Case—Improvement in Existing Reservoir: Increased Functionality of 25%		
Reservoir Duration (Hours)		
1	Minimum Reservoir Duration	0.6820
2	Mean Reservoir Duration	24.9466
3	Maximum Reservoir Duration	44.8640
Reservoir Utilization		
4	Minimum Reservoir Utilization	1.69%
5	Mean Reservoir Utilization	44.48%
6	Maximum Reservoir Utilization	95.93%
Ocean Region Duration (Hours)		
7	Minimum Region Duration	4.7356
8	Mean Region Duration	22.1191
9	Maximum Region Duration	49.9972
Ocean Region Utilization		
10	Minimum Region Utilization	3.32%
11	Mean Region Utilization	42.63%
12	Maximum Region Utilization	90.24%
Second Case–Improvement in Existing Reservoir : Increased Functionality of 50%		
Reservoir Duration (Hours)		
S/N	Parameter	Value
13	Minimum Reservoir Duration	0.3740
14	Mean Reservoir Duration	20.4409
15	Maximum Reservoir Duration	43.0199
Reservoir Utilization		
16	Minimum Reservoir Utilization	0.25%
17	Mean Reservoir Utilization	46.21%
18	Maximum Reservoir Utilization	89.81%
Ocean Region Duration (Hours)		
19	Minimum Region Duration	0.7464
20	Mean Region Duration	23.6839
21	Maximum Region Duration	49.0802
Ocean Region Utilization (%)		
22	Minimum Region Utilization	4.48%
23	Mean Region Utilization	51.21%
24	Maximum Region Utilization	93.51%
Third Case–Improvement in Existing Reservoir : Increased Functionality of 75%		
Reservoir Duration (Hours)		
S/N	Parameter	Value
25	Minimum Reservoir Duration	3.9682
26	Mean Reservoir Duration	23.0112
27	Maximum Reservoir Duration	43.5903
Reservoir Utilization (%)		
28	Minimum Reservoir Utilization	5.54%
29	Mean Reservoir Utilization	53.28%
30	Maximum Reservoir Utilization	91.97%
Ocean Region Duration (Hours)		
31	Minimum Region Duration	2.0323
32	Mean Region Duration	27.6452
33	Maximum Region Duration	47.6819
Ocean Region Utilization (%)		
34	Minimum Region Utilization	1.14%
35	Mean Region Utilization	43.38%
3426	Maximum Region Utilization	96.34%

The simulation also considers that each of the considered ocean regions has a varying capacity to provide support for hosting and ensuring the continued functionality of UDC. Such a consideration accommodates the heterogeneity and the dynamics since ocean regions have different operational capabilities.

**Figure 9:**
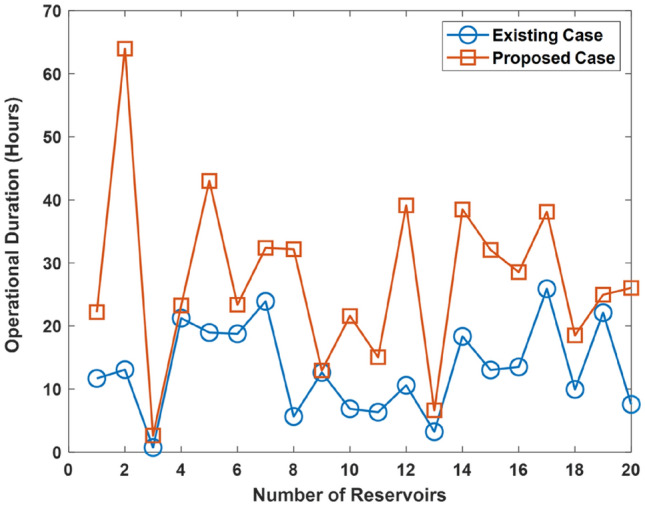
Operational duration in the case where there is improvement in the functionality of the existing reservoir by 25%.

**Figure 10:**
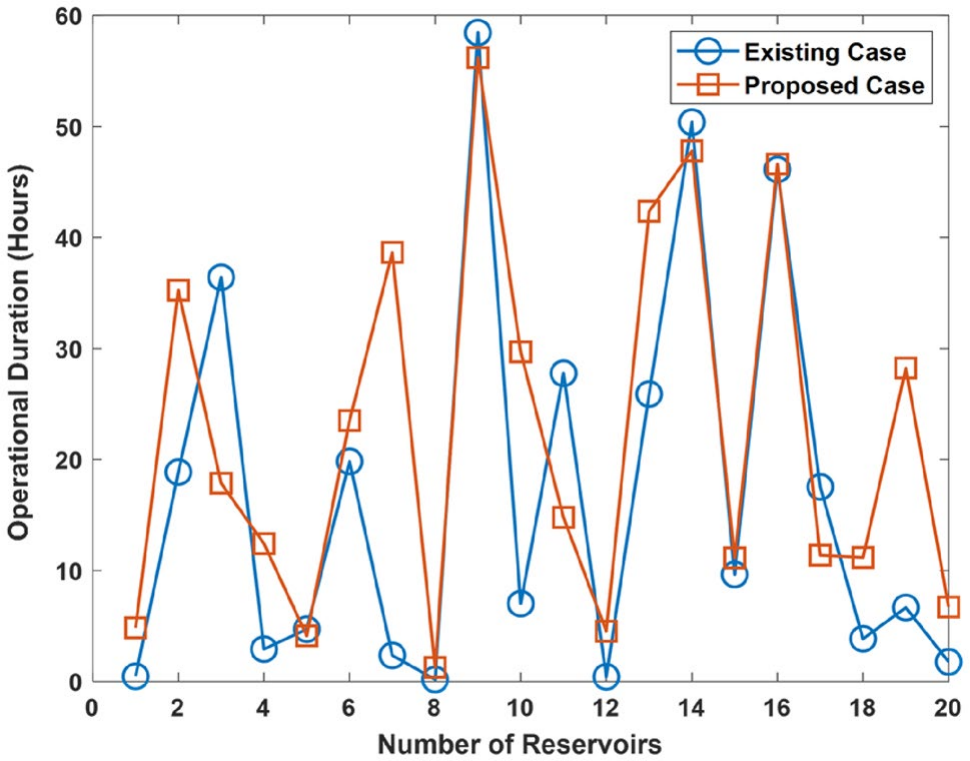
Operational duration in the case where there is improvement in the functionality of the existing reservoir by 50%.

**Figure 11:**
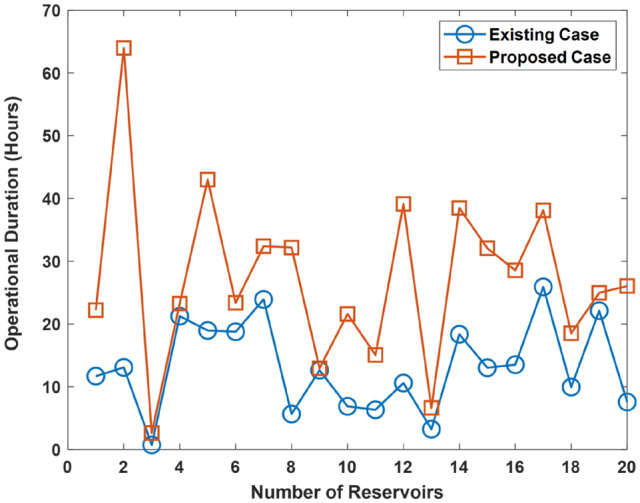
Operational duration in the case where there is improvement in the functionality of the existing reservoir by 75%.

Results showing the duration for the case of the improvement in the reservoir functionality by 25, 50, and 75% is shown in Figs. 9, 10, and 11, respectively. The results show that the proposed approach improves UDC operational duration. This shows that the incorporation of reservoir diversity feature in the proposed approach serves to enhance the UDC operational duration i.e., uptime. The improvement arises due to the reservoir’s ability to provide a supply of cold water. The cold water being supplied is used for cooling the UDC during warming events that leads to a rise in the ocean temperature. However, an improvement in the reservoir functionality increases the operational duration of the existing UDC.

Further analysis shows that the operational duration is enhanced by an average of 48.5, 28.8, and 8.9% due to the incorporation of the proposed approach. In the case concerned, the functionality of the existing reservoir is improved by 25, 50, and 75%, respectively. Therefore, the operational duration is enhanced by an average of 8.9–48.5% due to the use of the proposed approach.

### Second aspect—evaluation and analysis of the overhead

**Figure 12:**
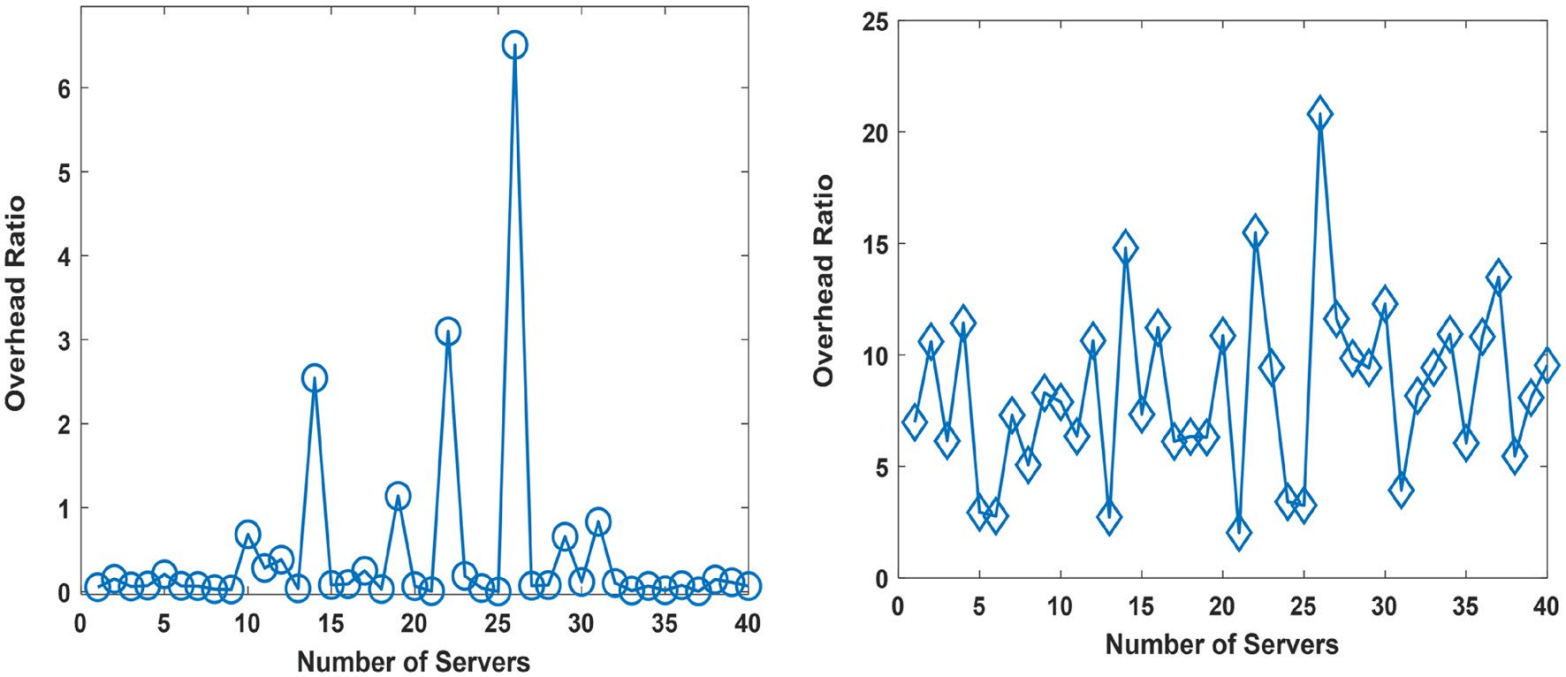
Overhead in Context 1 for the Proposed Approach (Right), and the Existing Approach (Left).

The performance evaluation focusing on the overhead is presented in this aspect. The overhead is evaluated for the cases of the existing approach, and proposed approach. In the existing and proposed approach, the overhead arises due to data requiring processing and arising from ocean environment observations. In our consideration, the monitoring of the ocean environment is data intensive and described using 100 environmental variables i.e., information elements. Each environmental variable is presented in a manner that the data size is 1 MB. The investigation is done for the existing case and proposed case in two contexts i.e., Context 1, and Context 2. The case in Context 1 is for the case where the UDC has 3 reservoirs. The case in Context 2 is for the case where the UDC has 6 reservoirs. The simulation parameters for Context 1 and Context 2 are in Tables 2, and 3, respectively. The performance simulation results for Context 1, and Context 2 are in Figs. 11, and 12, respectively.

**Table 2:** Context 1: Simulation Parameters.

S/N	Parameter	Value
Server Capacity		
1	Number of Servers in the Underwater Data Center (UDC)	40
1	Minimum Server Computing Capacity	0.1086 GB
2	Mean Server Computing Capacity	5.0060 GB
3	Maximum Server Computing Capacity	9.5560 GB
Proportion of Computing Resources for Marine Heat Waves Monitoring Per Reservoir		
4	Number of Reservoir per Underwater Data Center (UDC)	3
4	Minimum Proportion of Server Capacity for Marine Heat Wave Monitoring	0.43%
5	Mean Proportion of Server Capacity for Marine Heat Wave Monitoring	47.76%
6	Maximum Proportion of Server Capacity for Marine Heat Wave Monitoring	99.17%
Proportion of Computing Resources for Underwater Volcanic Event Monitoring Per Reservoir		
7	Minimum Proportion of Server Capacity for Underwater Volcanic Event Monitoring	0.45%
8	Mean Proportion of Server Capacity for Underwater Volcanic Event Monitoring	15.86%
9	Maximum Proportion of Server Capacity for Marine Heat Wave Monitoring	33.3%
Proportion of Computing Resources for Additional Event Monitoring Per Reservoir		
10	Minimum Proportion of Server Capacity for Additional Event Monitoring	0.371%
11	Mean Proportion of Server Capacity for Additional Event Monitoring	24.45%
12	Maximum Proportion of Server Capacity for Additional Event Monitoring	49.70%
Computing Resources available on Autonomous Underwater Vehicles (AUVs)		
13	Number of Autonomous Underwater Vehicles (AUVs)	10
13	Minimum Computing Resources available aboard AUVs	8.275 MB
14	Mean Computing Resources available aboard AUVs	473.8 MB
15	Maximum Computing Resources available aboard AUVs	966.7 MB

**Table 3:** Simulation Parameters for the Case in Context 2.

S/N	Parameter	Value
Server Capacity		
1	Number of Servers in the Underwater Data Center (UDC)	40
1	Minimum Server Computing Capacity	0.8999 GB
2	Mean Server Computing Capacity	5.5656 GB
3	Maximum Server Computing Capacity	9.8051 GB
Proportion of Computing Resources for Marine Heat Waves Monitoring Per Reservoir		
4	Number of Reservoir per Underwater Data Center (UDC)	6
4	Minimum Proportion of Server Capacity for Marine Heat Wave Monitoring	7.52%
5	Mean Proportion of Server Capacity for Marine Heat Wave Monitoring	53.2%
6	Maximum Proportion of Server Capacity for Marine Heat Wave Monitoring	92.6%
Proportion of Computing Resources for Underwater Volcanic Event Monitoring Per Reservoir		
7	Minimum Proportion of Server Capacity for Underwater Volcanic Event Monitoring	%
8	Mean Proportion of Server Capacity for Underwater Volcanic Event Monitoring	8.814%
9	Maximum Proportion of Server Capacity for Marine Heat Wave Monitoring	16.585%
Proportion of Computing Resources for Additional Event Monitoring Per Reservoir		
10	Minimum Proportion of Server Capacity for Additional Event Monitoring	0.03067%
11	Mean Proportion of Server Capacity for Additional Event Monitoring	12.43%
12	Maximum Proportion of Server Capacity for Additional Event Monitoring	25.79%
Computing Resources available on Autonomous Underwater Vehicles (AUVs)		
13	Number of Autonomous Underwater Vehicles (AUVs)	10
13	Minimum Computing Resources available aboard AUVs	3.656 MB
14	Mean Computing Resources available aboard AUVs	504.64 MB
15	Maximum Computing Resources available aboard AUVs	998.20 MB

Figures 11, and 12 present the overhead ratio evaluation results for the proposed approach and existing approach on the left and right, respectively. In Fig. 11, the proposed approach reduces the overhead in comparison to the existing approach. The overhead ratio describes the proportion of computing resources expended on executing ocean environment related monitoring and manoeuvring activities. In the simulation, the server computing capacity is of the capacity of Gigabytes. The server computing capacity in this case is deemed low. However, the choice of this computing capacity has been done to enable the determination of the overhead ratio in a manner where the results have an improved clarity. The clarity of the results is enhanced for an overhead ratio in the range shown compared to small numerical values of the order of × 10^–2^ which may be obtainable in the case of high server capacity.

**Figure 13:**
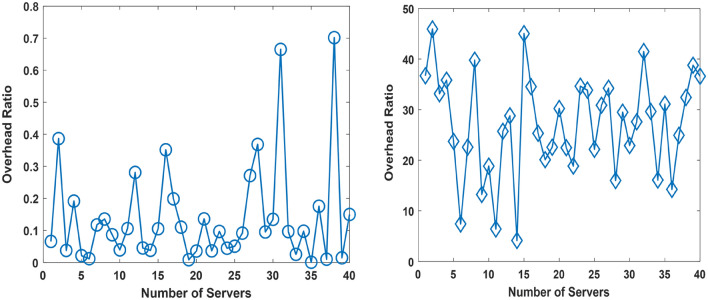
Overhead in Context 2 for the Proposed Approach (Right), and the Existing Approach (Left).

A comparison of results obtained in the Existing Approach is presented on the right in Figs. 12, and 13. The presented results shows that the overhead ratio increases with the number of reservoirs aboard the UDC. Further analysis shows that using the proposed approach in context 1, and context 2 reduces the overhead ratio by 95.8, and 99.4%, respectively. Therefore, the proposed approach reduces the overhead by 95.8–99.4% on average.

### Third aspect—PUE evaluation and analysis

**Figure 14:**
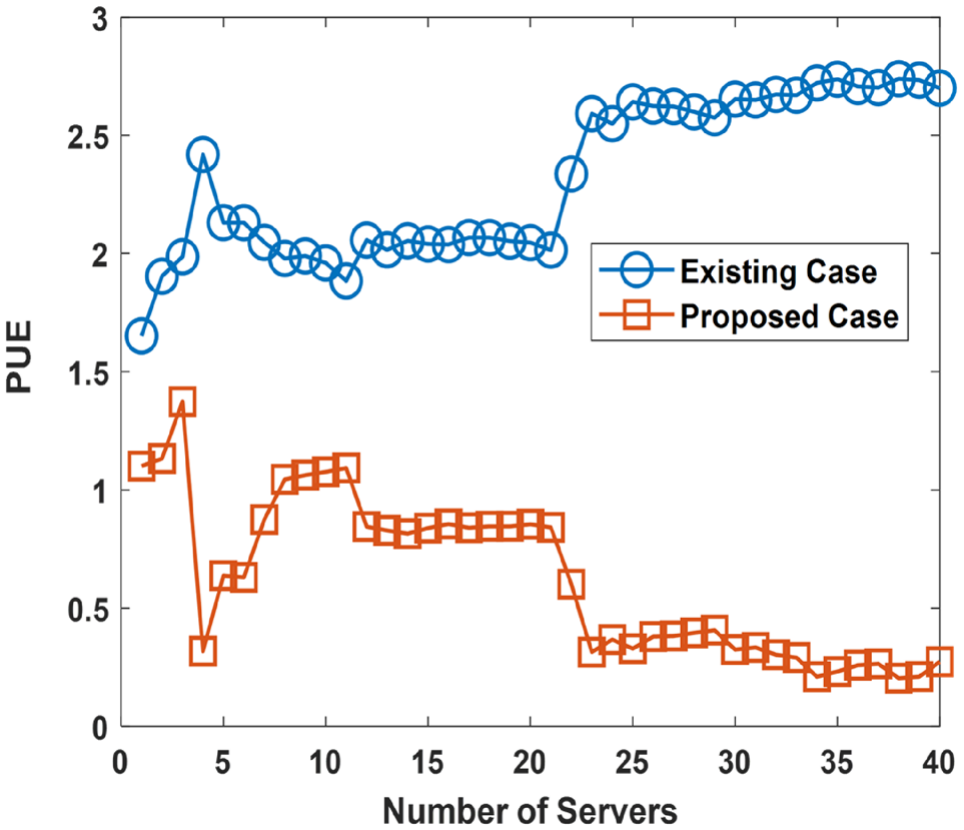
PUE Performance in Case 1.

**Figure 15:**
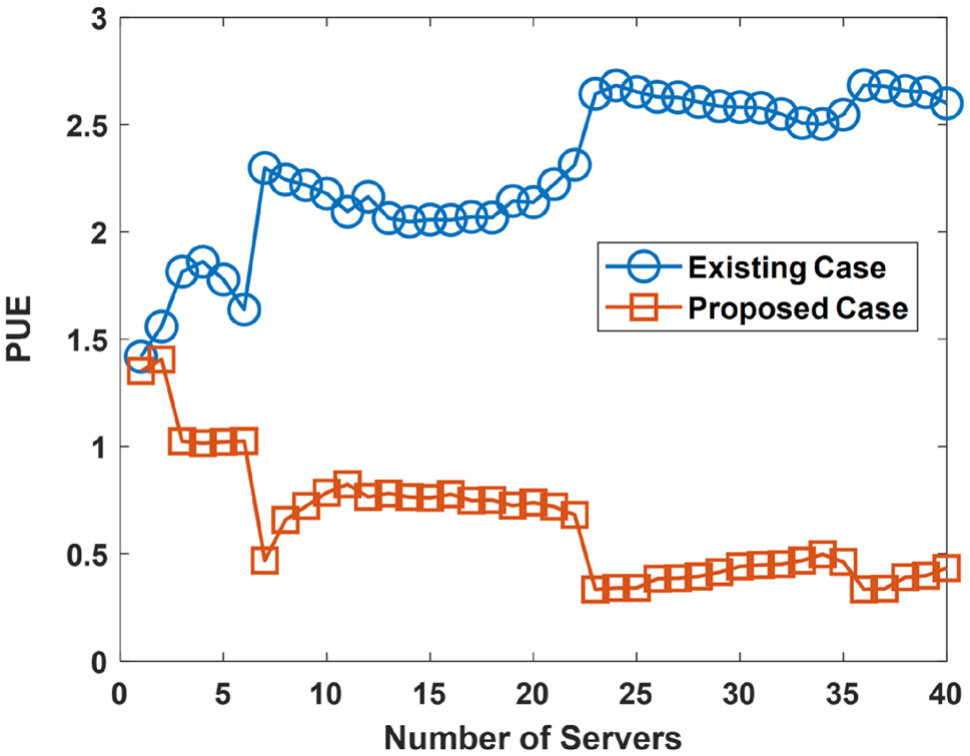
PUE Performance in Case 2.

An evaluation of the PUE is presented in this aspect. The investigation of the PUE is done with the goal of determining how the proposed approach enhances the PUE. A low PUE is more beneficial than a higher PUE. The parameters used for the PUE simulation are presented in Table 4. The PUE simulation considers three cases with each incorporating the occurrence of ocean warming increasingly. This is described by the ocean cooling factor (which is increasing) across each of the considered cases. Ocean warming occurrence increases from Case 1 to Case 2, and to Case 3. In the scenario associated with Case 1, Case 2, and Case 3; the ocean warming is at 10, 30, and 50% on average, respectively. The evaluation results are presented for Case 1in Fig. 13, Case 2 in Fig. 14, and Case 3 in Fig. 15. The considered UDC utilize two-socket servers with varying levels of utilization. The two-socket server has a power consumption of 365 W^64^.

**Table 4:** PUE Investigation: Simulation Parameters.

S/N	Parameter	Value
Case 1—Ocean Warming is at 10% on Average		
1	Minimum Server Power Consumption	8.81 W
2	Mean Server Power Consumption	163.07 W
3	Maximum Server Power Consumption^46^	332. 66 W
4	Minimum Proportion of Power Spent Cooling	0.32%
5	Mean Proportion of Power Spent Cooling	49.5%
6	Maximum Proportion of Power Spent Cooling	98.4%
7	Minimum Power Spent in Server Cooling	71.46 W
8	Mean Power Spent in Server Cooling	7.31 kW
9	Maximum Power Spent in Server Cooling	26.67 kW
10	Minimum Ocean Cooling Factor	0.0728%
11	Mean Ocean Cooling Factor	4.86%
12	Maximum Ocean Cooling Factor	9.84%
13	Minimum Proportion of Power used by Reservoir	0.305%
14	Mean Proportion of Power used by Reservoir	5.94%
15	Maximum Proportion of Power used by Reservoir	12.42%
16	Minimum Reservoir Power	12.91 W
17	Mean Reservoir Power	1.02 kW
18	Maximum Reservoir Power	3.61 kW
Case 2—Ocean Warming is at 30% on Average		
19	Minimum Server Power Consumption	8.81 W
20	Mean Server Power Consumption	163.07 W
21	Maximum Server Power Consumption	332. 66 W
22	Minimum Proportion of Power Spent Cooling	0.57%
23	Mean Proportion of Power Spent Cooling	54. 6%
24	Maximum Proportion of Power Spent Cooling	99.2%
25	Minimum Power Spent in Server Cooling	67.5 W
26	Mean Power Spent in Server Cooling	8.64 kW
27	Maximum Power Spent in Server Cooling	28.84 kW
28	Minimum Ocean Cooling Factor	0.019%
29	Mean Ocean Cooling Factor	15. 57%
30	Maximum Ocean Cooling Factor	29.85%
31	Minimum Proportion of Power used by Reservoir	0.25%
32	Mean Proportion of Power used by Reservoir	5.87%
33	Maximum Proportion of Power used by Reservoir	12.50%
34	Minimum Reservoir Power	10.06 W
35	Mean Reservoir Power	973.24 W
36	Maximum Reservoir Power	3.92 kW
Case 3—Ocean Warming is at 50% on Average		
37	Minimum Server Power Consumption	8.81 W
38	Mean Server Power Consumption	163.07 W
39	Maximum Server Power Consumption	332. 66 W
40	Minimum Proportion of Power Spent Cooling	3.141%
41	Mean Proportion of Power Spent Cooling	56.93%
42	Maximum Proportion of Power Spent Cooling	99.21%
43	Minimum Power Spent in Server Cooling	76.47 W
44	Mean Power Spent in Server Cooling	10.2 kW
45	Maximum Power Spent in Server Cooling	30.9 kW
46	Minimum Ocean Cooling Factor	0.163%
47	Mean Ocean Cooling Factor	25.2%
48	Maximum Ocean Cooling Factor	47.1%
49	Minimum Proportion of Power used by Reservoir	0.016%
50	Mean Proportion of Power used by Reservoir	5.41%
51	Maximum Proportion of Power used by Reservoir	11.82%
52	Minimum Reservoir Power	3.06 W
53	Mean Reservoir Power	822.16 W
54	Maximum Reservoir Power	2.42 kW

**Figure 16:**
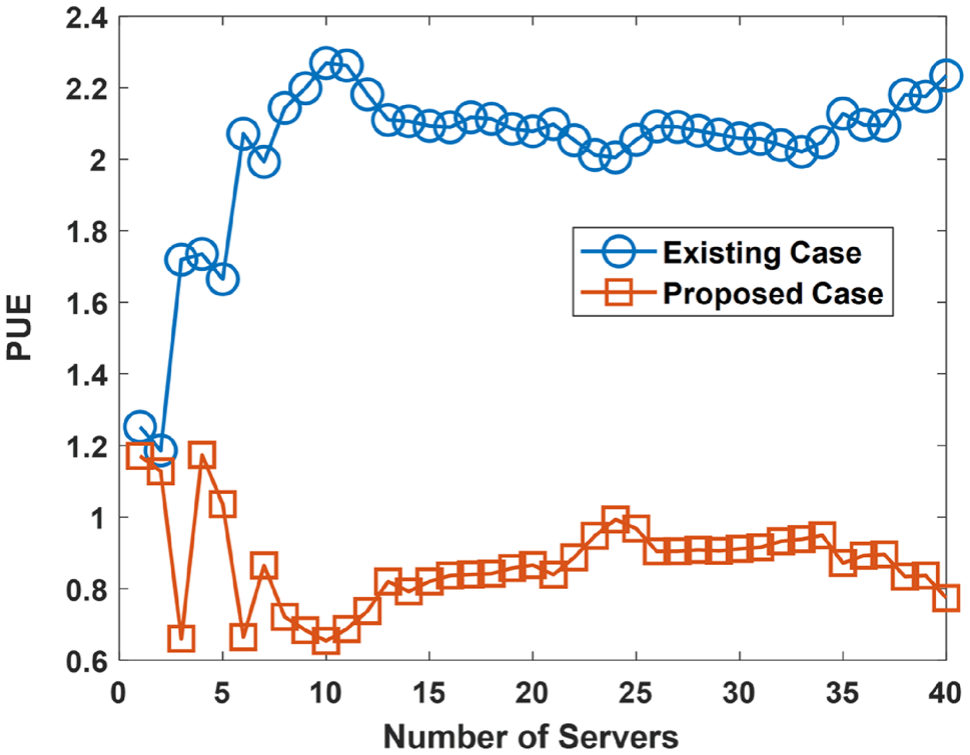
PUE Performance in Case 3.

The results presented in Figs. 14, 15, and 16 shows that the use of the proposed approach improves the PUE (low PUE figures are better than high PUE figures). Analysis shows that using the proposed approach instead of the existing approach in Case 1, Case 2, and Case 3, enhances the PUE. The PUE is enhanced by an average of 70.7, 69, and 55.6%, for Case 1, Case 2, and Case 3 respectively. A reduction in the PUE related performance improvement has arisen in the simulation due to the consideration of the occurrence of ocean warming in an increasing manner in the simulation.

## Conclusion

The presented research addresses the challenge of ensuring continued underwater data center cooling and functioning. The limiting effects of marine heat waves and underwater volcanoes are recognized to affect underwater data center cooling performance. The research recognizes that the use of reservoirs is non–scalable due to high computing and data overhead. The proposed solution presents a mobile and intelligent underwater data center capable of migration. The proposed solution presents a mobile underwater data center that executes migration. The migration is from an ocean region having high level of underwater ground displacement to one having low level or zero detected underwater ground displacement. The proposed data center also utilizes a dynamic approach with a service level agreement for application support. In addition, the presented research discusses approaches that enable service providers to deploy services without having to launch and operate own underwater data center systems. Evaluation shows that the use of the proposed mobile underwater data center improves the operational duration, and the power usage effectiveness by an average of 8.9–48.5%, and 55.6–70.7%, respectively. The use of the mobile underwater data center also reduces the overhead by an average of 95.8–99.4%.

## Data Availability

All data generated or analysed during this study are included in this published article [and its supplementary information files].

## References

[1] Haghshenas K, Setz B, Blosch Y, Aiello M (2023). Enough hot air: The role of immersion cooling. Energy Inform..

[2] Buyya R, Ilager S, Arroba P (2023). Energy-efficiency and sustainability in new generation cloud computing: A vision and directions for integrated management of data centre resources and workloads. Softw. Pract. Exp..

[3] Joshi Y, Kumar P (2012). Energy Efficient Thermal Management of Data Centers.

[4] Sethuramalingam, R. *et al.* Energy demand reduction in data centres using computational fluid dynamics. In *Springer Proceedings in Energy* 275–284. 10.1007/978-3-031-30960-1_26 (2023).

[5] Hoosain MS, Paul BS, Kass S, Ramakrishna S (2022). Tools towards the sustainability and circularity of data centers. Circ. Econ. Sustain..

[6] Zhang Q, Yang S (2021). Evaluating the sustainability of big data centers using the analytic network process and fuzzy TOPSIS. Environ. Sci. Pollut. Res..

[7] Zhang Y, Liu J (2022). Prediction of overall energy consumption of data centers in different locations. Sensors.

[8] Mao L, Chen R, Cheng H, Lin W, Liu B, Wang JZ (2023). A resource scheduling method for cloud data centers based on thermal management. J. Cloud Comput..

[9] Manganelli M, Soldati A, Martirano L, Ramakrishna S (2021). Strategies for improving the sustainability of data centers via energy mix, energy conservation, and circular energy. Sustainability.

[10] Periola AA (2023). Power usage effectiveness in stratosphere based computing platforms: Re-evaluation and need to re-establish performance benefits via an intelligent light server selection architecture. Wirel. Pers. Commun..

[11] Au Y (2022). Data centres on the Moon and other tales: A volumetric and elemental analysis of the coloniality of digital infrastructures. Territ. Polit. Gov..

[12] Periola A, Alonge A, Ogudo K (2020). Space habitat data centers—for future computing. Symmetry.

[13] Limón, R. Data centers move into space to mitigate power consumption and pollution. EL PAÍS English. https://english.elpais.com/science-tech/2022-12-28/data-centers-move-into-space-to-mitigate-power-consumption-and-pollution.html (2022).

[14] Periola AA, Alonge AA, Ogudo KA (2020). Architecture and system design for marine cloud computing assets. Comput. J..

[15] Periola AA, Alonge AA, Ogudo KA (2022). Heat wave resilient systems architecture for underwater data centers. Sci. Rep..

[16] Logan, K. *et al.* Improving data centre efficiency in Ireland. Available: https://energyinstitute.ucd.ie/wp-content/uploads/2020/12/Data-Centres_Insights-Series.pdf (2020).

[17] Hu G, Li L, Ren Z, Zhang K (2023). The characteristics of the 2022 Tonga volcanic tsunami in the Pacific Ocean. Nat. Hazards Earth Syst. Sci..

[18] Lopatka A (2021). A new undersea volcano is born east of Africa. Phys. Today.

[19] Dar F (2022). Upscaling fog computing in oceans for underwater pervasive data science using low-cost micro-clouds. ACM Trans. Internet Things.

[20] Hu Z-H, Zheng Y-X, Wang Y-G (2022). Packing computing servers into the vessel of an underwater data center considering cooling efficiency. Appl. Energy.

[21] Sutherland T, Bopp G (2023). The Pacific futures of subsea data centers. New Media Soc..

[22] Kadambi, Y., Vishal, R. & Ravish, R. Monitoring and alert systems for underwater data centers using Arduino. In *2021 IEEE International Conference on Computation System and Information Technology for Sustainable Solutions (CSITSS), 2021*. 10.1109/csitss54238.2021.9683449 (2021).

[23] XiaoyunGuang C, Liu WQu, Zhao Z (2023). Dynamic data collection algorithm based on mobile edge computing in underwater internet of things. J. Cloud Comput..

[24] Wang B, Ben K, Hua L, Zuo M, Zhang F (2022). EP-ADTA: edge prediction-based adaptive data transfer algorithm for underwater wireless sensor networks (UWSNs). Sensors.

[25] Luo P (2022). Efficient underwater sensor data recovery method for real-time communication subsurface mooring system. J. Mar. Sci. Eng..

[26] Juza M, Fernández-Mora À, Tintoré J (2022). Sub-Regional marine heat waves in the Mediterranean Sea from observations: Long-term surface changes, Sub-surface and coastal responses. Front. Mar. Sci..

[27] Chen W, Staneva J, Grayek S, Schulz-Stellenfleth J, Greinert J (2022). The role of heat wave events in the occurrence and persistence of thermal stratification in the southern North Sea. Nat. Hazards Earth Syst. Sci..

[28] Rosselló P, Pascual A, Combes V (2023). Assessing marine heat waves in the Mediterranean Sea: A comparison of fixed and moving baseline methods. Front. Mar. Sci..

[29] Lavayssière, S., Bazin, S., Royer, J.-Y. & Raumer, P.-Y. Hydroacoustic monitoring of Mayotte underwater volcanic eruption. 10.5194/egusphere-egu23-7141 (2023).

[30] Choi H, Kim SS, Park SH, Kim HJ (2021). Geomorphological and spatial characteristics of underwater volcanoes in the easternmost Australian-Antarctic Ridge. Remote Sens..

[31] Nam K, Wang F, Yan K, Zhu G (2023). Characteristics and time-series monitoring by GOES-17 of volcano flume on 15 January 2022 from Tonga submarine volcanic eruption. Geoenviron. Disasters.

[32] Gómez-Letona M, Arístegui J, Ramos AG, Montero MF, Coca J (2018). Lack of impact of the El Hierro (Canary Islands) submarine volcanic eruption on the local phytoplankton community. Sci. Rep..

[33] Dede B, Priest T, Bach W, Walter M, Amann R, Meyerdierks A (2023). High abundance of hydrocarbon-degrading Alcanivorax in plumes of hydrothermally active volcanoes in the South Pacific Ocean. ISME J..

[34] Wan Z (2022). The thermal effect of submarine mud volcano fluid and its influence on the occurrence of gas hydrates. J. Mar. Sci. Eng..

[35] Aiuppa A, Hall-Spencer JM, Milazzo M, Turco G, Caliro S, Di Napoli R (2020). Volcanic CO_2_ seep geochemistry and use in understanding ocean acidification. Biogeochemistry.

[36] Hansen J (2016). Ice melt, sea level rise and superstorms: Evidence from paleoclimate data, climate modeling, and modern observations that 2 °C global warming could be dangerous. Atmos. Chem. Phys..

[37] Newland EL, Mingotti N, Woods AW (2022). Dynamics of deep-submarine volcanic eruptions. Sci. Rep..

[38] Voosen, P. ‘It’s just mind boggling. More than 19,000 undersea volcanoes discovered. www.science.org. https://www.science.org/content/article/it-s-just-mind-boggling-more-19-000-undersea-volcanoes-discovered. Accessed 19 Apr 2023.

[39] Nalewicki, J. ‘Mind boggling’ array of 19,000 undersea volcanoes discovered with high-resolution radar satellites. livescience.com. https://www.livescience.com/planet-earth/rivers-oceans/mind-boggling-array-of-19000-undersea-volcanoes-discovered-with-high-resolution-radar-satellites (2023). Accessed 20 Oct 2023.

[40] Watson, C. Almost 20,000 Ancient Volcanoes Discovered at The Bottom of The Ocean. ScienceAlert. https://www.sciencealert.com/almost-20000-ancient-volcanoes-discovered-at-the-bottom-of-the-ocean (2023). Accessed 20 Oct 2023.

[41] G. I. Team. More Than 19,000 Volcanoes Discovered at the Bottom of the Ocean, Geology In. https://www.geologyin.com/2023/05/more-than-19000-volcanoes-discovered-at.html. Accessed 20 Oct 2023.

[42] Loy, S. 43,000 underwater volcanoes and counting. https://indianapublicmedia.org/amomentofscience/43,000-underwater-volcanoes-and-counting.php (2023). Accessed 20 Oct 2023.

[43] Astronuc. New Seamount discovered off coast of California,” Physics Forums: Science Discussion, Homework Help, Articles. https://www.physicsforums.com/threads/new-seamount-discovered-off-coast-of-california.1051023/ (2023). Accessed 20 Oct 2023.

[44] Morganti TM (2022). Giant sponge grounds of Central Arctic seamounts are associated with extinct seep life. Nat. Commun..

[45] Seismological Society of America. Machine Learning Reveals Magma Movement at Axial Seamount | Seismological Society of America. www.seismosoc.org. https://www.seismosoc.org/news/machine-learning-reveals-magma-movement-at-axial-seamount/. Accessed 20 Oct 2023.

[46] Herrera S (2023). From basalt to biosphere: Early non-vent community succession on the erupting Vailulu’u deep seamount. Front. Mar. Sci..

[47] Gevorgian J, Sandwell DT, Yu Y, Kim S, Wessel P (2023). Global distribution and morphology of small seamounts. Earth Space Sci..

[48] McPhee, R. New technique doubles the number of underwater mountains discovered» Explorersweb. Explorersweb. https://explorersweb.com/20000-underwater-mountains-discovered/ (2023). Accessed 20 Oct 2023.

[49] Ansari, A. How Microsoft kept its underwater datacenter connected while retrieving it from the ocean. Inside Track Blog. https://www.microsoft.com/insidetrack/blog/how-microsoft-kept-its-underwater-datacenter-connected-while-retrieving-it-from-the-ocean/ (2020).

[50] Steers, S. Why is Subsea Cloud putting data centres underwater? datacentremagazine.com. https://datacentremagazine.com/data-centres/why-is-subsea-cloud-putting-data-centres-underwater (2022).

[51] Madaline. D Highlander completes first underwater facility and looks to develop net-zero wind-powered data center. baxtel.com. https://baxtel.com/news/highlander-completes-first-underwater-facility-and-looks-to-develop-net-zero-wind-powered-data-center. Accessed 20 Oct 2023.

[52] Zhou M (2022). Analysis of the anomalous environmental response to the 2022 Tonga volcanic eruption based on GNSS. Remote Sens..

[53] Kilburn CRJ, Bell AF (2022). Forecasting eruptions from long-quiescent volcanoes. Bull. Volcanol..

[54] Seropian G, Kennedy BM, Walter TR, Ichihara M, Jolly AD (2021). A review framework of how earthquakes trigger volcanic eruptions. Nat. Commun..

[55] Jayakody DNK, Garg S, Kaddoum G, Shamim Hossain M (2022). Dual-hop mixed FSO-VLC underwater wireless communication link. IEEE Trans. Netw. Serv. Manag..

[56] Ali MF, Jayakody DNK (2023). SIMO-underwater visible light communication (UVLC) system. Comput. Netw..

[57] Ali MF, Perera TDP, Jayakody DNK (2022). O2O: An underwater VLC approach in Baltic and North Sea. Electronics.

[58] Ali, M. F., Jayakody, D. N., Ribeiro, M. V. A hybrid UVLC-RF and optical cooperative relay communication system. 10.1109/iciafs52090.2021.9606069 (2021).

[59] Aman, W., Al-Kuwari, S., Kumar, A., Rahman, M. M. & Muzzammil, M. Underwater and air-water wireless communication: state-of-the-art, channel characteristics, security, and open problems. Cornell University. 10.48550/arxiv.2203.02667 (2022).

[60] Razzaq A, Mohsan SAH, Li Y, Alsharif MH (2023). Architectural framework for underwater IoT: Forecasting system for analyzing oceanographic data and observing the environment. J. Mar. Sci. Eng..

[61] Jahanbakht M, Xiang W, Hanzo L, Azghadi MR (2021). Internet of underwater things and big marine data analytics—A comprehensive survey. IEEE Commun. Surv. Tutor..

[62] Qian C, Huang B, Yang X, Chen G (2021). Data science for oceanography: From small data to big data. Big Earth Data.

[63] Wen J, Yang J, Jiang B, Song H, Wang H (2021). Big data driven marine environment information forecasting: A time series prediction network. IEEE Trans. Fuzzy Syst..

[64] Zodhya. How much power do the servers consume? Medium. https://medium.com/@zodhyatech/how-much-power-do-the-servers-consume-8f9620be71a (2022).

